# Integrating *in vitro* breeding, BLUP prediction, and marker analysis to enhance rice yield, quality, and blast resistance

**DOI:** 10.3389/fpls.2025.1588427

**Published:** 2025-09-16

**Authors:** Samah M. Abdelkhalek, Mohamed Abdelrahman, Tahany M. Mazal, Itoh Kimiko, Mostafa Elshenawy, Samah Aamer, Amr A. Hassan, Kotb A. Attia, Megahed Ammar

**Affiliations:** ^1^ Biotechnology Lab, Rice Research and Training Center, Field Crops Research Institute, Agricultural Research Center, Kafrelsheikh, Egypt; ^2^ Plant breeding and genetics laboratory, Joint FAO/IAEA Center of Nuclear Techniques in Food and Agriculture, International Atomic Energy Agency, Seibersdorf, Austria; ^3^ Institute of Science and Technology, Niigata University, Ikarashi-2, Nishiku, Niigata, Japan; ^4^ Gene Bank Lab, Rice Research and Training Center, Field Crops Research Institute, Agricultural Research Center, Kafrelsheikh, Egypt; ^5^ Grain Quality Lab, Rice Research and Training Center, Field Crops Research Institute, Agricultural Research Center, Kafrelsheikh, Egypt; ^6^ Rice Pathology Department, Plant Pathology Research Institute, Agricultural Research Center, Kafrelsheikh, Egypt; ^7^ Center of Excellence in Biotechnology Research, King Saud University, Riyadh, Saudi Arabia

**Keywords:** genetic gain, speed breeding, anther culture, double haploid lines, grain quality, sustainability, blast disease, BLUP

## Abstract

Rice is acting a critical role in global food security, being a staple food for more than half of the world’s population. *In vitro*-derived lines possess a significant opportunity to develop new plant material in a shorter time frame compared to conventional breeding. In the current study, we developed new rice genotypes *via in vitro* culture for enhanced yield, quality, and blast disease resistance. Significant differences were observed among the newly developed genotypes compared with the commercial cultivars for various vegetative and yield traits. The results indicated notable improved yield performance, quality, and blast resistance for the *in vitro*-developed lines. Furthermore, the selection of the top 5% of the genotypes resulted in a predicted genetic gain of 0.19 kg m^-²^ for grain yield, representing a 20.88% improvement over the genotypes’ mean yield of 0.91 kg m^-²^. Best linear unbiased prediction (BLUP) modeling for the studied traits was applied to identify the best-performing genotypes. Principal component analysis-based BLUP estimates identified two *in vitro*-derived lines, AC-2286 and AC-2729, as the best-performing *in vitro* genotypes. Both lines have higher yielding ability compared to the local cultivars; however, only AC-2286 was blast resistant under artificial inoculation and natural conditions. Interestingly, marker–trait association revealed that AC-2729 carries the favorable marker allele for grain yield, RM224-152bp, on chromosome 11 with a highly significant phenotypic effect (33%), while AC-2286 has more resistance ability to blast disease owing to its genetic background that carries several favorable blast-resistant alleles RM6887-152bp, RM224-165bp, RM13-151bp, and RM1370-165bp with high significant phenotypic effect (62%, 47%, 47%, and 31%, respectively). These findings increase the potential of the *in vitro*-derived lines for enhancing rice productivity, quality, and disease resistance in a few years compared to classic breeding, which provides valuable insights for future breeding programs.

## 1 Introduction

Rice (*Oryza sativa*) is the main source of calories for more than half of the people living on earth. It is the most strategic crop that contributes to the economics of several developing countries (Khush, 2005; Samal et al., 2022). Moreover, the demand for rice is increasing to meet the overgrowing world population and better living standards. Rice demand will significantly increase, as the growth rate of rice consumption in West Asia and Sub-Saharan Africa will increase by about 3% and 4% per year by 2030 (OECD, Food and Agriculture Organization of the United Nations, 2015). This demand increase is challenged by decreasing land and water resources as well as various biotic and abiotic stresses (Ghazy et al., 2023).

Classic rice breeding has successfully been used to develop several high yielding rice cultivars that are resilient to biotic and abiotic stresses (Abdelrahman et al., 2021; Selim et al., 2024). However, this approach requires six to nine selfing cycles, followed by three to five seasons of field evaluation to develop a new variety (Lantos et al., 2022). The rapid changes in climate and evolving genetic behavior of pathogens invading rice crop outpace the long time required with traditional breeding. With advancement in molecular biology and biotechnology, it is now feasible to mine, dissect, and accumulate favorable alleles in target genotypes. One key application of rice biotechnology is *in vitro* culture, an approved technique that significantly shortens the breeding cycle by enabling the fixation of homozygosity (Ali et al., 2021).

Anther culture is a powerful tool in rice breeding, genetics, and biotechnology, enabling the rapid development of new plant genotypes with desirable traits by achieving homozygosity in a single generation from the haploid plants (Mishra and Rao, 2016; Sun et al., 2023; Chen et al., 2024; Sahana et al., 2024). This technique significantly reduces the time and costs required to generate pure lines in rice. Alternatively, mature embryo culture is another *in vitro* technique that induces genetic variation in rice. It offers distinct advantages, including year-round availability and ability to be conveniently stored for extended periods (Ge et al., 2006; Chadipiralla et al., 2020). With proper manipulations of the explant type, culture medium, and growth conditions, it is possible to obtain embryogenic callus with a high regeneration capacity which is an essential prerequisite for the genetic improvement of rice (Vega et al., 2009). The use of *in vitro* culture can facilitate the development of genetically diverse rice lines (Wu et al., 2024) (Ali et al., 2021), blast-resistant genotypes (Purwoko et al., 2010; Islam et al., 2023), and rice genotypes with better grain quality (Pattnaik et al., 2020).

Rice blast, caused by the fungus *Magnaporthe oryzae* (*M. oryzae*), is a devastating rice disease that significantly causes yield losses and poses a serious threat to global rice production (Younas et al., 2023). Developing resistant genotypes is considered the most effective and economical strategy to control rice blast. However, high genetic variability and rapid evolution of *M. oryzae* populations (Sheoran et al., 2021) necessitate the utilization of advanced breeding tools that accelerate the development of newly developed genotypes against diverse pathogen races collected from diverse locations.

High grain quality and disease resistance are the primary prerequisites for releasing new rice cultivars. The rice market is strongly driven by grain quality traits, as consumer preference and acceptance determine the economic value of a variety (Dalton, 2004; Wang et al., 2012; Abdelrahman and Zhao, 2020). In Egypt, farmers particularly prefer bold japonica-type grains and blast-resistant cultivars (Abdelrahman et al., 2021). To effectively harness the potential of rice improvement and selection among genotypes, it is essential to evaluate the genetic performance across multiple traits. Statistical tools such as best linear unbiased prediction (BLUP) and principal component analysis (PCA) have proven invaluable in estimating genotypic values and exploring trait relationships within diverse breeding populations. BLUP allows the identification of superior genotypes by predicting their true genetic merit genotypes (Piepho et al., 2008; Slater et al., 2014; Vineeth et al., 2022), while PCA aids in visualizing multivariate trait variation and identifying the most influential traits (Charles, 2024). These methods are particularly useful when selecting for complex traits like grain yield, heading date, and plant height, which are influenced by both genetic and environmental factors (Slater et al., 2014; Olivoto et al., 2019; Sood et al., 2020; Abdelrahman et al., 2022; Ghazy et al., 2023). Therefore, incorporating BLUP and PCA into the evaluation framework enhances the precision of selection and informs breeding strategies aimed at developing improved rice lines harboring genes related to stress resistance. Marker–trait association is a powerful tool to identify genomic regions linked to high genotypic performance. Single marker analysis (SMA) is considered one of the tools that is used to identify the association between marker allele and the trait in concern. It has been successfully implemented in different plant population types and crops to identify several marker alleles linked to the trait under study (Zhang et al., 2009; Zongo et al., 2017).

In the current study, we aimed to develop high-quality and blast-resistant, true breeding lines using speed-breeding-based *in vitro* culture technology. To achieve this goal, several F_1_ hybrids were generated to enhance both the grain quality and blast resistance of different Egyptian rice cultivars. These hybrids were further advanced *via* androgenesis to produce a true breeding double haploid line (DHL). Furthermore, additional mature embryo culture-derived lines (ECL) were generated. These *in vitro*-generated lines were evaluated for grain yield, quality attributes, and blast resistance. We further studied genetic gain and heritability of the studied traits in the *in vitro*-derived rice lines, followed by BLUP and PCA analysis to identify the best-performing lines. Moreover, SMA was conducted to identify alleles significantly associated with key agronomic traits in the newly developed lines.

## 2 Results

### 2.1 Vegetative growth and yield performance of the *in vitro*-derived lines

**Figure 1:**
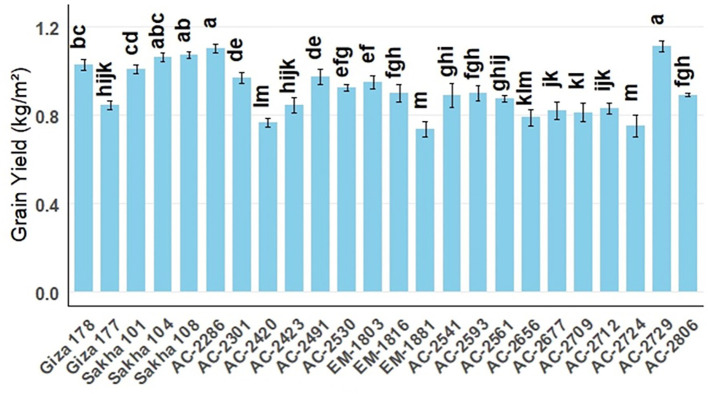
Average grain yield (kg m^-^²) of the newly developed *in vitro*-derived rice lines compared with five Egyptian commercial cultivars (Giza178, Giza177, Sakha101, Sakha104, and Sakha108), all grown over two successive seasons under identical field conditions. The blue bars show the average yields; the error bars denote ± SE. Different lowercase letters above the bars indicate statistically significant differences among genotypes.

**Figure 2:**
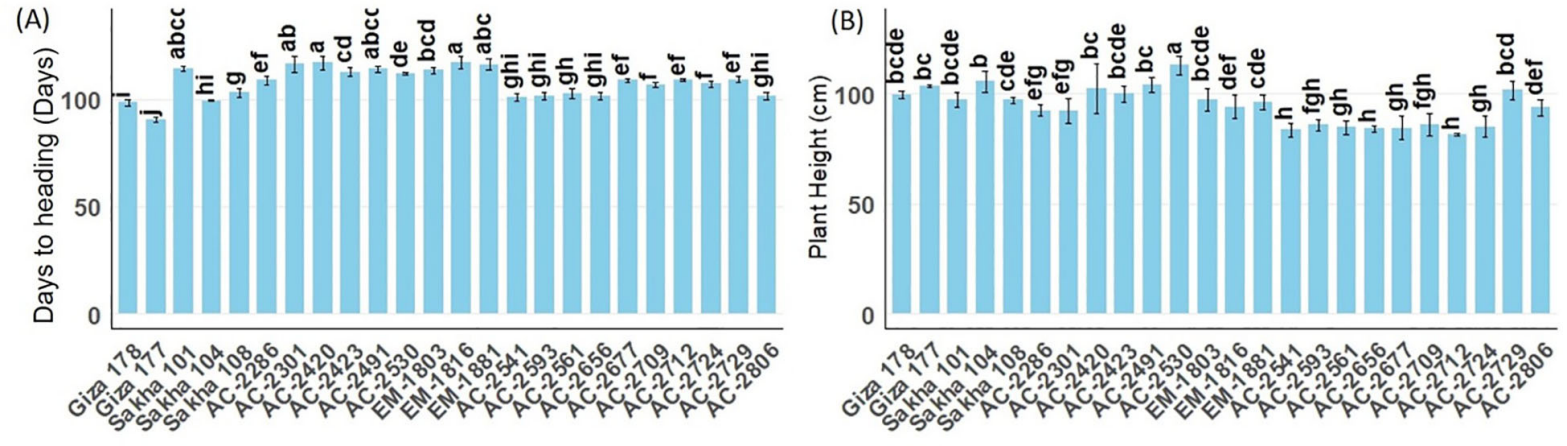
Mean days to heading [**(A)** and plant height (in cm, **(B)**] of the newly developed *in vitro*-derived rice lines compared with five Egyptian commercial cultivars (Giza 178, Giza 177, Sakha 101, Sakha 104, and Sakha 108). All genotypes were grown for two successive seasons under field conditions. The bars represent the mean ± SE, and different lowercase letters above each bar denote statistically significant differences among genotypes.

Highly significant differences were observed among the tested genotypes and the newly developed lines for the different vegetative and yield characteristics. Among the evaluated *in vitro*-derived lines, AC-2286 and AC-2729 emerged as the highest-yielding genotypes (**Supplementary Table S1**; **Figure 1**). Vegetative growth characteristics are essential for supporting plant performance and achieving high yield. In the current study, days to heading and plant height were considered (**Supplementary Table S1**; **Figure 2**). The high-yielding *in vitro* lines AC-2286 and AC-2729 exhibited average heading dates (109.00 and 109.33 days, respectively) compared to the earliest and latest commercial genotypes, Giza177 and Sakha101 (90.67 and 114.33, respectively). The best-performing *in vitro* line was AC-2541 which had 101.00 days to heading, indicating a whole life cycle of approximately 130 days. Regarding plant height, AC-2286 was featured with a short-stature structure (92.4 cm), whereas AC-2729 had an average plant height (101.47 cm). Notably, AC-2712 was the shortest among the newly developed lines (81.27 cm). Among the commercial genotypes, Sakha108 was the highest-yielding cultivar, with a short stature (96.93 cm) and a moderately long period to the heading date (103.33 days).

AC-2286 and AC-2729 recorded the highest average number of panicles per plant (24.33 and 24.00 panicles, respectively) exceeding the highest commercial cultivar, Sakha 108 (24 panicles). Those *in vitro*-derived lines have also had the best records for 1,000 grain weight (28.46 and 28.5 g, respectively). The correlation analysis revealed a highly significant relationship between yield and number of panicles per plant followed by 1,000 grain weight (*r* = 0.75 and 0.56, respectively), indicating that these traits play a crucial role in yield improvement.

### 2.2 The newly developed *in vitro*-derived lines’ quality characteristics

The mean performance of the genotypes for hulling, milling, head rice, kernel elongation, gelatinization, and amylose content percentages are presented in **Supplementary Table S2**. The results indicate that the genotypes exhibited statistically significant differences for all the quality characteristics studied (**Supplementary Table S3**).

#### 2.2.1 Grain physical properties of the *in vitro*-derived lines

**Figure 3:**
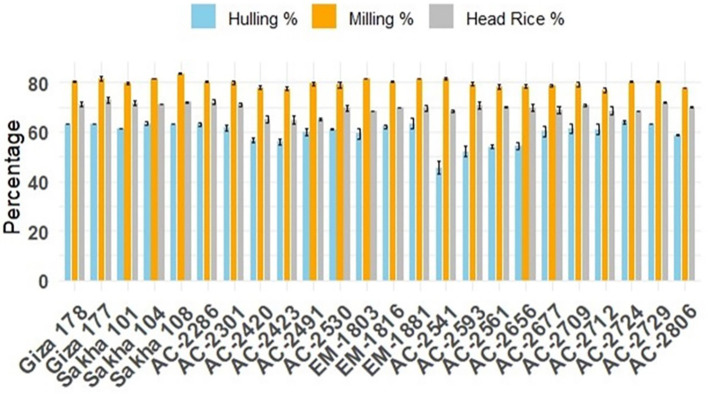
Average physical quality characteristics, hulling percentage (light blue), milling percentage (orange), and head-rice percentage (gray) of the newly developed *in vitro*-derived rice compared with the Egyptian cultivars (Giza178, Giza177, Sakha101, Sakha104, and Sakha108), all grown over two successive seasons under identical field conditions. The bars indicate the group means, and the error bars denote ± SE.

Dehulling is a crucial post-harvest process in rice production as high hulling percentages improve rice recovery. In the current study, the hulling percentage of the commercial cultivars ranged from 79.56% (Sakha101) to 83.56% (Sakha108). Among the newly developed lines, AC-2541 recorded a hulling percentage of 81.55% exceeding the average of the commercial cultivars. The differences in hulling percentage among AC-2286, AC-2729, Giza177, and Sakha104 (80.22%, 80.22%, 81.67%, and 81.56%, respectively) were not significant (**Figure 3**). For milling percentage, Giza177 has the highest milling percentage, 73.06%. However, the differences between this percentage and those of milled AC-2286, AC-2729, and AC-2806 (72.17%, 71.83%, and 71.83%) were not statistically significant. Interestingly, AC-2724 had the highest head rice percentage (64%) among all of the genotypes studied. This percentage was statistically similar to those of EM-1881, AC-2729, and AC-2286 (63.44%, 63.22%, and 63.11%, respectively).

#### 2.2.2 Grain chemical properties of the *in vitro*-derived lines

The cooking and eating quality of rice genotypes depend on the chemical properties of the grains. Gelatinization temperature (GT) directly influences cooking quality by determining the energy and time required for cooking. GT was estimated as the alkali spreading value (ASV) that is estimated by the extent of dispersal of whole milled rice grains in dilute alkali solution (1.7% KOH). The commercial cultivars exhibited low GT estimates ranging from 6.00 (for Sakha101, Sakha104, and Sakha108) to 6.33 (for Giza177 and Giza178). Among *in vitro*-derived lines, AC-2491 had the lowest GT estimate (2.00), whereas AC-2806 had the highest (7.00). These genotypes require the cooking temperature to be 55°C–74°C.

**Figure 4:**
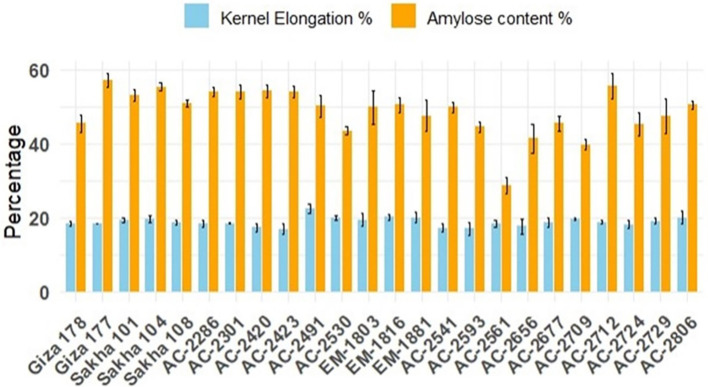
Average chemical quality characteristics, kernel-elongation percentage (blue bars), and amylose content percentage (orange bars) of the newly developed *in vitro*-derived lines grown for two successive seasons compared with the Egyptian cultivars (Giza178, Giza177, Sakha101, Sakha104, and Sakha108). The bars represent the mean values, and the error bars denote ± SE.

Furthermore, the commercial rice cultivars demonstrated relatively consistent kernel elongation percentages, with Giza 177 standing out as having the highest elongation. In contrast, the *in vitro*-derived lines displayed a broader range of elongation percentages, indicating greater variability. While some lines, such as AC-2712, achieved kernel elongation percentages (55.48%) comparable to those of the commercial cultivars (57.17%), others, like AC-2561, showed significantly lower elongation (28.68%). This variability highlights the potential for selecting *in vitro*-derived lines with desirable elongation characteristics. Amylose content is one of the key traits considered for selecting rice varieties prior to releasing. Among the commercial varieties, Giza 178 has the lowest amylose content percentage (18.38%). AC-2286 (18.27%), AC-2420 (17.32%), AC-2423 (16.81%), AC-2541 (17.21%), AC-2593 (16.95%), AC-2561 (18.28%), AC-2656 (17.58%), and AC-2724 (18.14) have estimates lower than that for Giza178 (18.38%) (**Figure 4**).

### 2.3 The newly developed *in vitro* lines resistant to blast disease

A total of 24 *M. oryzae* isolates were collected from Kafrelsheikh, Dakahlia, Gharbia, Sharkia, Beheira, and Damietta governorates during the 2023 and 2024 rice growing seasons (**Supplementary Table S4**). The isolates underwent race identification test, which resulted in their classification under the four blast race groups; IC, ID, IA, and II (**Supplementary Table S5**). These races were subsequently used for the artificial inoculation of the 24 rice genotypes under greenhouse conditions to assess their resistance to blast disease. Among the commercial rice cultivars, Sakha101 and Sakha104 were the most vulnerable to blast infection. In contrast, no infection was observed in the following *in vitro*-derived lines; AC-2286, AC-2301, AC-2420, AC-2423, AC-2541 AC-2593, AC-2561, AC-2656, AC-2677, AC-2709, AC-2712, AC-2724, and AC-2806 across all tested races (**Table 1**). To further validate these findings, we further conducted natural field evaluation at the blast nurseries in Sakha, Gemmiza, and Zarzora. The results indicated that the commercial cultivars Giza177 and Giza178 were resistant under natural conditions. Similarly, several *in vitro*-derived lines exhibited resistance to blast disease under natural field conditions: AC-2286, AC-2301, AC-2420, AC-2423, AC-2530, AC-2541, AC-2593, AC-2561, AC-2656, AC-2677, AC-2709, AC-2712, AC-2724, and AC-2806 (**Table 2**). These results suggest that several of the newly developed *in vitro*-derived lines possess high levels of resistance to blast disease, making them promising candidates for breeding programs aimed at improving blast resistance in rice.

**Table 1:** Reaction of 24 rice genotypes inoculated with the Egyptian tested races of *Magnaporthe oryzae* under greenhouse conditions.

No.	Genotypes	Race number/ race group																							
		EG1	EG2	EG3	EG4	EG5	EG6	EG7	EG8	EG9	EG10	EG11	EG12	EG13	EG14	EG15	EG16	EG17	EG18	EG19	EG20	EG21	EG22	EG23	EG24
		ID-16	IC-2	IC-20	IC-25	ID-12	ID-11	ID-15	IC-1	ID-11	IC-3	IC-15	ID-12	ID-16	IA-105	IC-15	IC-32	IC-32	ID-9	ID-15	II	IC-3	IC-3	IC-5	IC-3
**1**	Giza 178	1	1	1	1	1	1	1	1	1	1	1	1	1	3	1	1	1	1	1	1	1	1	1	1
**2**	Giza 177	1	1	1	1	1	1	1	1	1	1	1	1	1	3	1	1	1	1	1	1	1	1	1	1
**3**	Sakha 101	4	4	1	4	1	1	1	7	1	3	1	4	5	9	4	7	1	7	9	9	9	7	7	7
**4**	Sakha 104	1	7	4	1	4	1	4	9	4	4	4	1	1	7	1	4	1	7	9	4	1	4	7	7
**5**	Sakha 108	1	1	1	1	1	1	1	1	7	1	1	1	4	5	1	4	1	1	1	1	4	1	7	7
**6**	AC-2286	1	1	1	1	1	1	1	1	1	1	1	1	1	1	1	1	1	1	1	1	1	1	1	1
**7**	AC-2301	1	1	1	1	1	1	1	1	1	1	1	1	1	1	1	1	1	1	1	1	1	1	1	1
**8**	AC-2420	1	1	1	1	1	1	1	1	1	1	1	1	1	1	1	1	1	1	1	1	1	1	1	1
**9**	AC-2423	1	1	1	1	1	1	1	1	1	1	1	1	1	1	1	1	1	1	1	1	1	1	1	1
**10**	AC-2491	1	1	1	1	1	1	1	4	1	1	1	1	1	1	1	1	1	1	3	1	1	1	1	1
**11**	AC-2530	1	7	1	1	1	1	1	4	1	1	1	1	1	1	1	1	1	1	1	1	1	1	1	1
**12**	EM-1803	1	1	1	1	1	1	1	1	1	1	1	1	4	9	7	7	1	7	9	7	1	1	7	9
**13**	EM-1816	1	4	1	1	1	1	1	4	1	1	1	1	4	9	7	7	1	7	9	7	1	1	4	9
**14**	EM-1881	1	9	1	1	1	1	1	4	1	1	1	1	4	9	4	6	1	7	9	7	1	1	4	9
**15**	AC-2541	1	1	1	1	1	1	1	1	1	1	1	1	1	1	1	1	1	1	1	1	1	1	1	1
**16**	AC-2593	1	1	1	1	1	1	1	1	1	1	1	1	1	1	1	1	1	1	1	1	1	1	1	1
**17**	AC-2561	1	1	1	1	1	1	1	1	1	1	1	1	1	1	1	1	1	1	1	1	1	1	1	1
**18**	AC-2656	1	1	1	1	1	1	1	1	1	1	1	1	1	1	1	1	1	1	1	1	1	1	1	1
**19**	AC-2677	1	1	1	1	1	1	1	1	1	1	1	1	1	1	1	1	1	1	1	1	1	1	1	1
**20**	AC-2709	1	1	1	1	1	1	1	1	1	1	1	1	1	1	1	1	1	1	1	1	1	1	1	1
**21**	AC-2712	1	1	1	1	1	1	1	1	1	1	1	1	1	1	1	1	1	1	1	1	1	1	1	1
**22**	AC-2724	1	1	1	1	1	1	1	1	1	1	1	1	1	1	1	1	1	1	1	1	1	1	1	1
**23**	AC-2729	1	5	7	1	4	1	1	7	4	4	1	1	4	1	1	1	1	1	1	1	9	7	7	9
**24**	AC-2806	1	1	1	1	1	1	1	1	1	1	1	1	1	1	1	1	1	1	1	1	1	1	1	1
**1-2 = resistant 3 = moderately resistant 4-6 = susceptible 7-9 = highly susceptible ** *Race identification based on **Supplementary Tables 4**, **5** and **9**																									

**Table 2:** Reaction of 24 rice genotypes to blast disease at three locations during the 2023 season under field conditions.

NO	Rice genotypes	Sakha	Gemmiza	Zerzora
**1**	Giza 178	1	1	1
**2**	Giza 177	1	1	1
**3**	Sakha 101	4	5	7
**4**	Sakha 104	4	5	4
**5**	Sakha 108	4	4	7
**6**	AC-2286	1	1	1
**7**	AC-2301	1	1	1
**8**	AC-2420	1	1	1
**9**	AC-2423	1	1	1
**10**	AC-2491	4	4	4
**11**	AC-2530	1	1	1
**12**	EM-1803	4	5	4
**13**	EM-1816	4	5	5
**14**	EM-1881	5	6	5
**15**	AC-2541	1	1	1
**16**	AC-2593	1	1	1
**17**	AC-2561	1	1	1
**18**	AC-2656	1	1	1
**19**	AC-2677	1	1	1
**20**	AC-2709	1	1	1
**21**	AC-2712	1	1	1
**22**	AC-2724	1	1	1
**23**	AC-2729	6	4	1
**24**	AC-2806	1	1	1
**1= resistant, 3 = moderately resistant, 4-6 = susceptible, 7-9 = highly susceptible.**				

### 2.4 Genetic variability, heritability, and selection potential of key agronomic traits in rice

The estimated broad-sense heritability (H^2^) values varied among the analyzed traits, ranging from 0.49 for amylose percentage to 0.93 for heading date (**Supplementary Table S6**). High heritability estimates were observed for heading date, yield, and head rice percentage (0.93, 0.93, and 0.92, respectively), indicating strong genetic control over these traits and suggesting their potential for selection in breeding programs. The genetic coefficient of variation (GCV) ranged from 1.93% (hulling percentage) to 79.95% (blast score), while the phenotypic coefficient of variation (PCV) was highest for blast score (90.54%) and lowest for hulling percentage (2.07%). The low GCV and PCV values for hulling and milling percentages indicate limited variability, suggesting that these traits might be influenced more by environmental factors or have already been stabilized through selection. Conversely, the high GCV and PCV for blast score suggest a high degree of variability, which may allow significant genetic improvement through selection. The genetic advance as a percentage of the mean (GA%) was highest for gelatinization temperature and yield (25.26 and 21.12, respectively), followed by kernel elongation percentage (16.40%), while the lowest GA% was recorded for hulling percentage (3.88%). Furthermore, the relatively high GA% values for yield and gelanization temperature together with kernel elongation percentage suggest that substantial genetic improvement can be achieved in these traits through selection.

### 2.5 Genotype selection based on BLUP and PCA analysis

**Figure 5:**
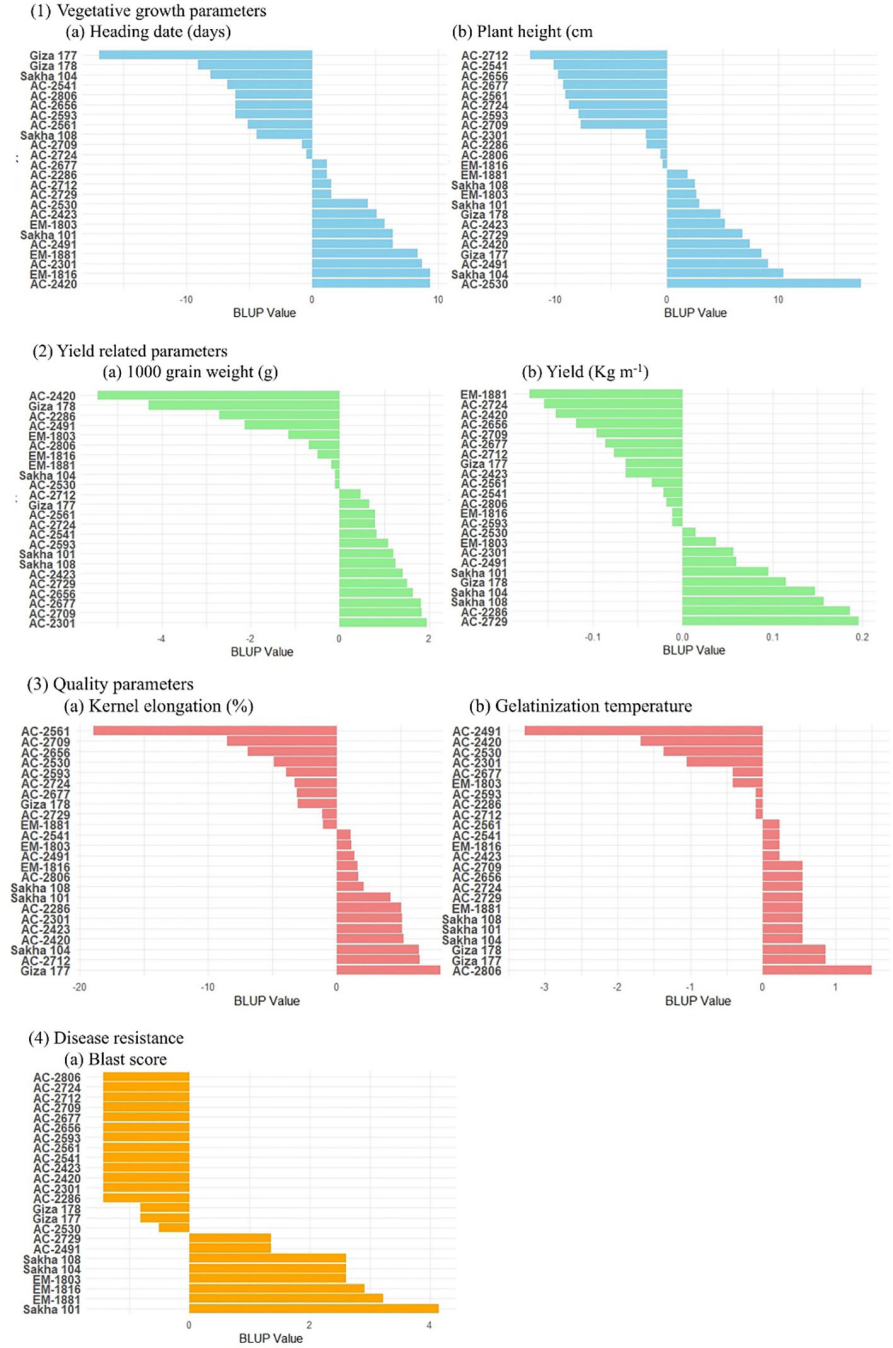
Ranking of the tested genotypes based on their BLUP estimates for selected studied traits: (1) vegetative traits, (2) yield related traits, (3) quality traits, and (4) blast disease response.

**Figure 6:**
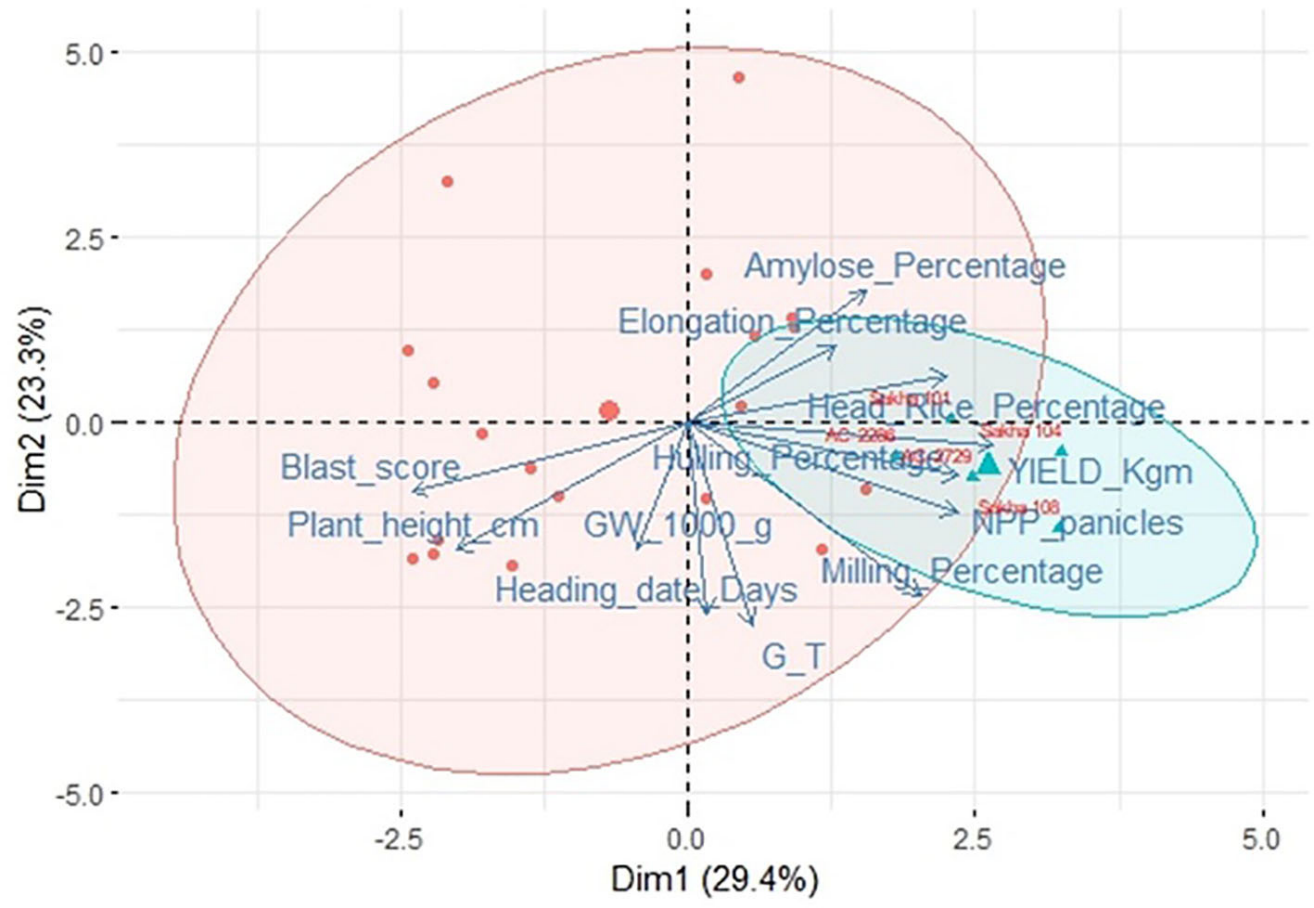
PCA biplot highlighting top genotypes based on their BLUP estimate for the different studied traits. Dim1 and Dim2 explain 29.4% and 23.3% of the total variation, respectively, capturing the major axes of trait diversity. The triangles represent top-performing genotypes (labeled in red) based on yield and milling-related and are enclosed within the blue ellipse. The red dots represent the remaining tested genotypes. Notably, genotypes AC-2286 and AC-2729 stand out due to their proximity.

The BLUP analysis was conducted to estimate the genetic performance of the 19 *in vitro*-derived rice lines compared with the five local varieties for the studied key agronomic traits (**Supplementary Table S7**, **Figure 5**). BLUP estimates of the *in vitro*-derived lines, AC-2541 and AC-2712, were the best for heading date and plant height compared to their counterpart lines (**Supplementary Table S7**), while for yield AC-2729 recorded the best estimate. At the same time, for quality traits, the *in vitro*-derived lines AC-2712, AC-2423, AC2541, AC2561, and AC2491 were the best for the hulling, milling, head rice percentage, kernel elongation percentage, gelatinization temperature, and amylose content, respectively, while for disease resistance, several *in vitro*-derived lines were resistant to blast disease (**Figure 5-4a**). To identify the best-performing genotype among the *in vitro*-derived lines, we conducted PCA analysis using the BLUP estimates for the whole genotypes for the different agronomic trait under study (**Figure 6**). The results of the PCA indicates that the first two principal components (Dim1 (29.4%) and Dim2 (23.3%)) capture a substantial portion of the variation (52.7%) in the data. The red cluster consists of those genotypes that exhibit moderate performance across the trait under study. Interestingly, the genotypes AC-2729 and AC-2286 were among the top five ranked genotypes and were comparable to the local ones Sakha104, Sakha108, and Sakha101. Furthermore, yield-related traits such as grain yield, number of panicle per plant, and milling percentage are grouped on the positive side of Dim1, while traits such as blast score, days to heading, and plant height are clustered on the negative side, indicating inverse relationships with yield.

### 2.6 Identification of SSR marker alleles associated with phenotypic data via single marker association analysis

To identify genomic regions in the tested genotypes that are significantly associated with the studied traits, single marker analysis was conducted (**Table 3**). For heading date, the marker alleles RM208-180bp and RM208-176bp located on chromosome 2 were significantly associated with *R* ^2^ = 0.44 for both alleles, while for plant height, the marker alleles RM248-80bp (chromosome 7), RM13-151bp, and RM13-135bp (chromosome 5) had phenotypic effect of 30%, 31%, and 31%, respectively. The alleles RM248-80bp and RM13-135bp were favorable alleles, contributing to the reduction in plant height by averages of -9.63 and -7.63 cm, respectively.

**Table 3:** Single marker analysis and allelic effect on the studied parameters.

Traits	Marker allele	Chr. No.	Allelic effect	R^2^	Type of allele	Traits	Marker allele	Chr. No.	Allelic effect	R^2^	Type of allele
HD (days)	RM208-180bp	2	-9.32	0.44**	Favorable allele	Head rice (%)	RM1216-150bp	1	-4.75	0.27**	Unfavorable allele
	RM208-176bp	2	9.32	0.44**	Unfavorable allele		RM1216-158bp	1	4.75	0.27**	Favorable allele
PH (cm)	RM248-80bp	7	-9.63	0.30**	Favorable allele		RM1368-165bp	6	-4.97	0.31**	Unfavorable allele
	RM13-151bp	5	7.63	0.31**	Unfavorable allele		RM1368-183bp	6	4.97	0.31**	Favorable allele
	RM13-135bp	5	-7.63	0.31**	Favorable allele		RM286-128bp	11	-6.75	0.51**	Unfavorable allele
PN (panicles)	RM5891-150bp	3	3.24	0.27**	Favorable Allele		RM3428-172bp	11	4.97	0.31**	Favorable allele
	RM235-130bp	1	3.88	0.29**	Favorable Allele		RM3428-145bp	11	-6.92	0.57**	Unfavorable allele
	RM1986-180bp	12	-3.84	0.45**	Unfavorable Allele	GT	RM3513-125bp	3	-1.39	0.30**	Unfavorable allele
1000-GW (g)	RM6787-152bp	1	-5.43	0.46**	Unfavorable allele	AC (%)	RM286-128bp	11	-1.48	0.32**	Unfavorable allele
	RM6787-169bp	1	5.43	0.46**	Favorable allele	Blast disease	RM6787-152bp	1	-4.36	0.62**	Favorable allele
	RM13-151bp	5	-4.89	0.37**	Unfavorable allele		RM6787-169bp	1	4.36	0.62**	Unfavorable allele
	RM13-135bp	5	4.89	0.37**	Favorable allele		RM13-151bp	5	-3.82	0.47**	Favorable allele
	RM286-115bp	11	5.05	0.40**	Favorable allele		RM13-135bp	5	3.82	0.47**	Unfavorable allele
GY (kg m^-2^)	RM224-152bp	11	0.17	0.33**	Favorable allele		RM224-165bp	11	-2.86	0.27**	Favorable allele
Hulling (%)	RM6787-152bp	1	-2.13	0.45**	Unfavorable allele		AP5930-168bp	6	-2.88	0.29**	Favorable allele
	RM6787-169bp	1	2.13	0.45**	Favorable allele		RM1341-175bp	11	-2.86	0.27**	Favorable allele
	AP5930-168bp	6	24.74	0.29**	Favorable allele		RM1341-197bp	11	2.86	0.46**	Unfavorable allele
Milling (%)	RM5891-150bp	8	2.74	0.29**	Favorable allele		RM1368-165bp	6	-3	0.31**	Favorable allele
Head rice (%)	RM5891-162bp	8	3.3	0.60**	Favorable allele		RM1368-183bp	6	3	0.31**	Unfavorable allele
	RM235-110bp	1	-8.25	0.57**	Unfavorable allele		RM3428-172bp	11	3	0.31**	Unfavorable allele

HD, days to heading; PH, plant height; PN, panicle per plant; 1,000-GW, 1,000 grain weight; GY, grain yield; GT, gelatinization temperature; AC, amylose content.

**Significant at the 0.01 significance level.

For panicle number per plant, significant associations were detected with the alleles RM5891-150bp, RM235-130, and RM1986-180bp (with *R* ^2^ values of 0.27, 0.29, and 0.45, respectively). Similarly, RM6887 and RM13 were significantly associated with 1,000 grain weight (*R* ^2^ = 0.46 and 0.37, respectively). Additionally, RM224-152bp was the only marker segment that expressed association (*R* ^2^ = 0.33) with grain yield. For quality traits, RM6887 showed a highly significant association with the hulling% (*R* ^2^ = 0.45), while the marker segment RM5891-150bp was associated with milling percentage (*R* ^2^ = 0.29), both having a positive effect on its percentage in the studied genotypes. RM3513-125bp was significantly associated (*R* ^2^ = 0.30) with genitalization temperature, while no marker alleles showed any association at 0.01 significance level for kernel elongation in our study. Interestingly, several marker alleles were significantly associated with blast resistance in this study. RM13 and RM6887 were highly associated with blast resistance in the studied genotypes exhibiting 0.47 and 0.62 coefficient of determination, respectively.

## 3 Discussion

In this study, we successfully developed and evaluated several *in vitro*-derived rice lines for their vegetative growth, yield performance, grain quality, and resistance to blast disease. Among the newly developed lines, AC-2286 and AC-2729 emerged as the highest-yielding genotypes, demonstrating superior vegetative characteristics and yield components. A significant correlation was detected between grain yield and the 1,000 grain yield, explaining the performance of the highest-yielding *in vitro* lines, AC-2286 and AC-2729, which recorded the highest 1,000 grain weight values. High-quality lines such as AC-2541 and AC-2724 exhibited notable improvement in grain quality traits, particularly hulling percentage and head rice recovery. Additionally, several lines (AC-2286, AC-2301, AC-2420, AC-AC-2423, AC-2530, AC-2541, AC-2593, AC-2561, AC-2656, AC-2677, AC-2709, AC-2712, AC-2729, and AC-2806) exhibited resistance to multiple physiological races of the blast pathogen. These findings highlight the potential of *in vitro*-derived lines in enhancing rice productivity, quality, and disease resistance in a shorter breeding cycle.

Our findings align with previous research demonstrating the potential of *in vitro* techniques, such as anther culture and mature embryo culture, to expedite the breeding process and enhance genetic diversity (Otani et al., 2005; Sun et al., 2023). Reducing the time required to develop new cultivars through these technologies is considered one of the important approaches for the sustainability of rice cultivation (Dwivedi et al., 2015). The significant differences observed in vegetative and yield traits among the *in vitro*-derived lines corroborate the effectiveness of these techniques in generating high-yielding rice cultivars (Lantos et al., 2022).

The superior grain quality traits observed in lines such as AC-2541 and AC-2724, particularly in terms of hulling percentage and head rice recovery, are consistent with the findings of Pattnaik et al. (2020), who reported the benefits of *in vitro* culture in improving grain quality traits. The variability in cooking quality traits among the *in vitro* lines further highlights the potential for selecting lines with desirable attributes, as noted in previous studies (Dalton, 2004; Wang et al., 2012). The resistance of several *in vitro*-derived lines to various races of *M. oryzae* aligns with the findings of Islam et al. (2023) and Younas et al. (2023), who emphasized the importance of developing blast-resistant rice genotypes through advanced biotechnological approaches.

Genetic gain is a key parameter for evaluating the progress and efficiency of a breeding program. It provides valuable insights for refining breeding methodologies and strategy, optimizing resource allocation, and accelerating the development of several improved varieties (Khanna et al., 2024). In this study, the selection of the top 5% of genotypes from those *in-vitro* lines resulted in a predicted genetic gain of 0.19 kg m^-^² for grain yield (**Supplementary Table S6**), representing a 20.88% improvement over the genotypic mean yield of 0.91 kg m^-^² (**Supplementary Table S1**). This considerable increase highlights the efficacy of the anther culture breeding method and selection approach and emphasizes the potential for further enhancement of yield through targeted breeding efforts. These findings further confirm that the development of high-yielding, high-quality, and blast-resistant rice lines through *in vitro* culture techniques has significant implications for rice breeding programs, particularly in regions facing biotic and abiotic stresses. The rapid generation of homozygous lines via anther culture and mature embryo culture significantly reduces the breeding cycle, enabling the timely development of improved cultivars to meet the increasing demand for rice (Tamilzharasi et al., 2024; Bančič et al., 2025). BLUP prediction of breeding value is a standard practice in plant breeding programs, allowing for the selection of superior individuals based on genetic merit. In this study, BLUP prediction model was able to select two anther-culture-derived lines among the top five genotypes, AC-2286, and AC-2729 as indicated in the PCA biplot (**Figure 6**, **Supplementary Table S8**). The positions of those genotypes in the PCA space in the right quadrant confirm their agronomic superiority compared to other *in vitro*-derived rice lines, reflecting a favorable combination of high yield potential, good panicle number, and superior grain processing quality. The identification of markers associated with yield components, grain quality, and disease resistance can facilitate marker-assisted selection, further enhancing the efficiency of rice breeding programs. SMA has the statistical power to detect the association between the marker segment and the phenotype. Early maturing genotypes are preferable by the breeders; the SSR marker fragment RM208 was highly associated with heading date in our studied genotypes.

Allele RM208-180bp has a negative effect on the heading date being the elite allele. With RM208 marker located on chromosome 2, previous reports confirmed the presence of QTL linked to heading date on chromosome 2 (Lin et al., 1998; Luo et al., 2005; Zhu et al., 2015). The marker alleles RM248-80bp and RM13-135bp on chromosomes 7 and 5, respectively, exhibited highly significant association with 30% and 31% coefficient of determination, causing a reduction of -9.63 and -7.63 in the plant height in our studied genotypes, respectively. Recently, Sitoe et al. reported favorable alleles linked to plant height on chromosome 5 in a natural population composed of 504 rice accessions (Sitoe et al., 2022). The favored marker alleles RM5891-150bp (chromosome 8) and RM235-130bp (chromosome 1) for number of panicles per plant were exhibited in the best-performing genotypes as these alleles have a positive impact on the genotype’s number of panicle per plant, at 3.24 and 3.88 panicles, respectively, while a negative effect was detected for a highly significant marker RM 1986-180bp (chromosome 12) that causes a reduction with about -3.84 panicles in number of panicles per plant. Zhu et al. reported a major QTL located on the long arm of chromosome 1 that was responsible for this phenotypic variation of the panicle number per plant (Zhu et al., 2011).

**Figure 7:**
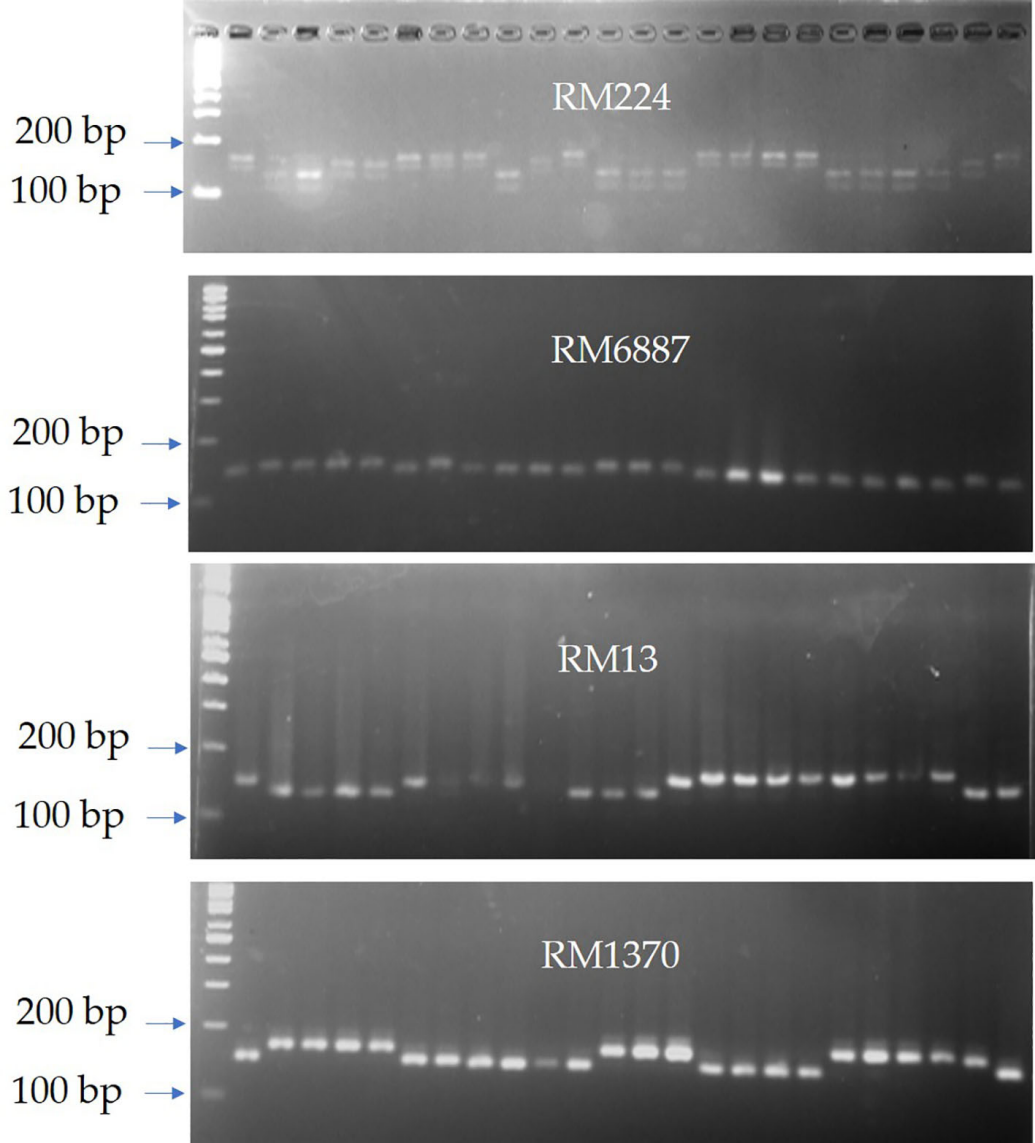
SSR banding patterns of the four microsatellite markers (RM224, RM6887, RM13, and RM1370) that showed high significant association with grain yield and blast resistance in the BLUP selected genotypes AC-2286 and AC-2729. Lane 1: 100-bp ladder; lanes 2–6: commercial Egyptian cultivars; lanes 7–25 are the newly developed *in vitro*-derived lines.

Interestingly, most of the *in vitro*-derived lines with high-quality estimates were harboring to marker alleles that are significantly linked to quality characteristics with positive effect on those characters. Among those alleles with positive effect are AP5930-170bp (24.74% on hulling), RM5891-150bp (2.74% on milling), and RM1216-158bp, RM1370-183bp, and RM3428-172bp (4.75%, 4.97%, and 4.97% on head rice percentage, respectively). Marker alleles that negatively affect blast disease is preferred by plant breeders as these alleles enhance disease resistance. RM224-165bp that is highly linked to blast resistance in our study was carried only in genotypes that are resistant to blast disease at all locations. Similar results are aligned with those obtained by Divya et al., confirming that the marker RM224 showed significant *R* ^2^ values for leaf blast, lesion type, lesion number, infected leaf area, and potential disease incidence percentage (Divya et al., 2014). Intriguingly, AC-2286 and AC-2729 are the best performing *in vitro*-derived genotypes. Both lines have higher yielding ability compared to the local cultivars. Interestingly, marker–trait association revealed that AC-2729 carries the favorable marker allele RM224-152bp on chromosome 11 with highly significant phenotypic effect (33%), while AC-2286 has more resistance ability to blast disease owing to its genetic background that carries several favorable alleles such as RM6887-152bp, RM13-151bp, RM1370-165bp, and RM224-165bp with a high significant phenotypic effect (62%, 47%, 31%, and 27%, respectively) (**Figure 7**). Similar results were obtained by Abdelrahman et al. (2021) when they applied SMA to a detected marker associated with grain yield performance of a set of 19 newly developed F_n_ rice genotypes compared with their parents.

Our results also underscore the importance of integrating molecular marker techniques, such as SSR marker analysis, to identify and select for desirable traits. The identification of markers associated with yield components, grain quality, and disease resistance can facilitate marker-assisted selection, further enhancing the efficiency of rice breeding programs. A major strength of this study is the comprehensive evaluation of *in vitro*-derived lines for a wide range of traits, including vegetative growth, yield performance, grain quality, and disease resistance. The use of multiple physiological races of blast to assess blast resistance adds robustness to our findings.

## 4 Materials and methods

### 4.1 Development of the *in vitro*-derived rice lines

**Figure 8:**
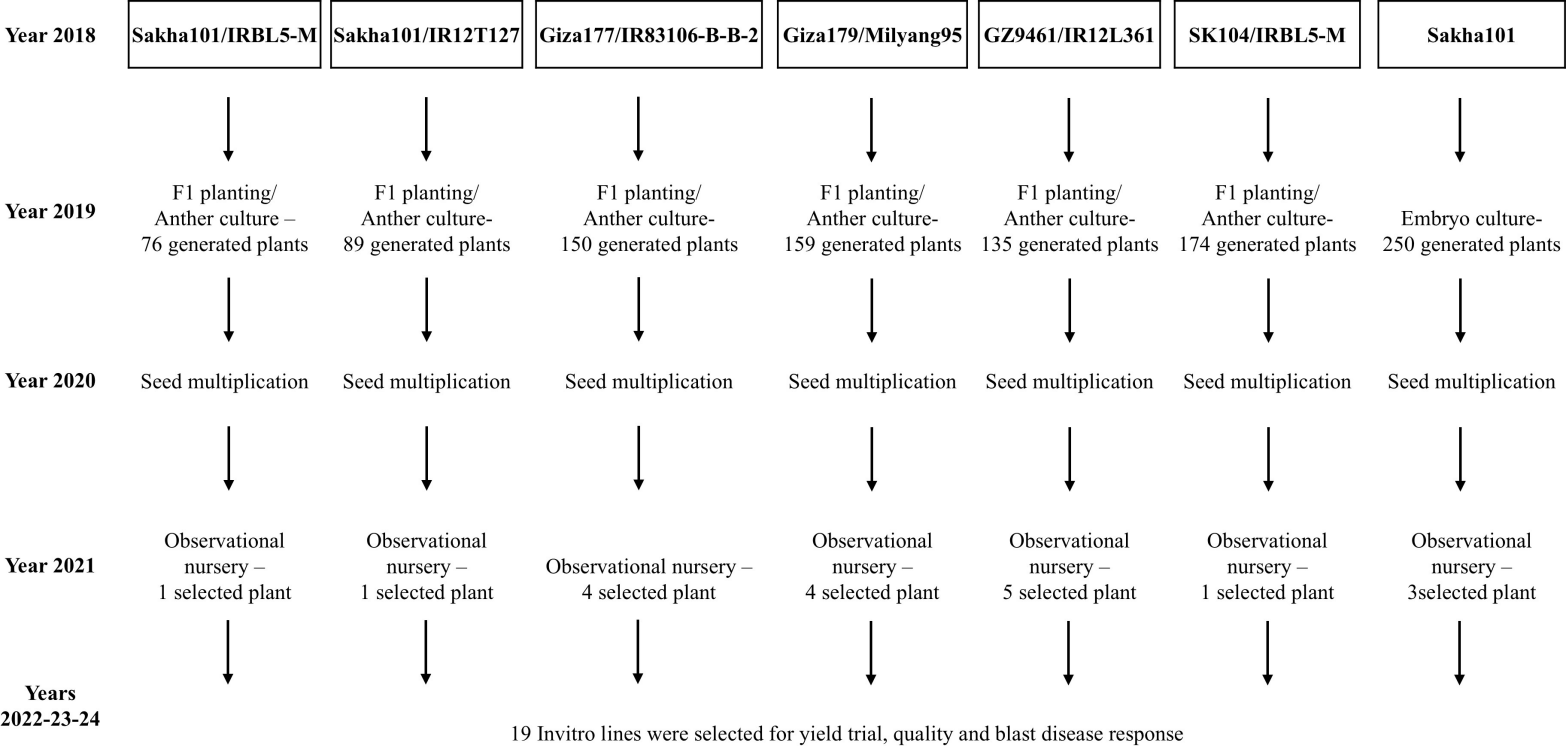
Flowchart representing the multi-year breeding and selection process for developing the 19 new rice lines through *in-vitro* techniques (2018–2023). The breeding scheme started in 2018 with the use of Egyptian parent varieties including Sakha101, Giza177, GZ9461, and Sakha104 together with the donor varieties IRBL5-M, IR12T127, IR83106-B-B-2, Milyang95, and IR12L361. Various breeding methods (F_1_ planting, anther culture, and embryo culture) were implemented in 2019, resulting in the generation of numerous plants. In 2020, seed multiplication was performed to increase plant populations. In 2021, observational nurseries were conducted to evaluate and select promising plants from each cross. The final step (2022–2023) selected 19 *in vitro*-derived lines that were evaluated for yield performance and quality and for blast disease resistance during 2023–2024.

Five Egyptian rice genotypes, Giza177, Giza179, Sakha101, Sakha104, and GZ9461 were used in this experiment to enhance their yield, quality, and/or blast resistance. The donor genotypes consisted of IRBL5-M, IR12T127, IR83106-B-B, Milyang95, and IR12L361. The seeds of the donor genotypes were collected from the genetic stock of rice research and training center, Sakha 33717, Egypt. Those genotypes were used to develop F_1_ hybrid needed for the current experiment, while the mature embryo of the cultivar Sakha101 was used to develop embryogenesis-derived lines as explained in the study scheme (**Figure 8**). The F_1_ hybrids were cultivated at the experimental farm of the Rice Research and Training Center in Sakha, Kafrelsheikh, Egypt. During the booting stage, healthy boots were collected from each genotype between 07:00 and 09:00 a.m. as described by Afza et al. (2000). The boots collection and pretreatment were conducted as described by Dash et al. (2022).

### 4.2 Anther and embryo plating and plant regeneration

The composition and media preparation, along with the detailed steps from anther and embryo culture to plant regeneration and adaption in the screenhouse, are provided in the **Supplementary Material** and methods file.

### 4.3 Field evaluation for the *in vitro*-derived lines

The seeds of the *in vitro*-derived lines were planted under observational nursery for a season based on their field performance. A total of 19 lines were selected. These 19 lines along with the most cultivated cultivars Giza178, Giza177, Sakha101, Sakha104, and Sakha108 being used as check varieties (**Table 1**) were cultivated for the two successive rice growing seasons 2022 and 2023. The field evaluation for these lines was conducted at the experimental farm of rice research and training center, Sakha Agricultural Research Station, Kafrelsheikh, Egypt (31°09′ N, 30°9′ E) in a randomized complete block design (RCBD) with three replications. The plots were five 1-m-long rows with planting space of 20 cm between transplants and 20 cm between rows. All other cultural practices were conducted as recommended by the Egyptian national rice crop program. The *in vitro*-derived lines’ phenotypic performance was evaluated by measuring the following: days to heading (days), plant height (cm), number of panicles per plant (panicle), 1,000-seed weight (g), and grain yield (kg m^-2^) according to the rice standard evaluation system (Rice, I.N.F.G.E.O, 1996). The *in vitro*-derived lines’ quality characteristics were further assessed by measuring the physical, cooking, and eating properties of grains at the grain quality lab, Rice Research and Training Center, Kafrelsheikh, Egypt. Hulling (%), milling (%), and head rice (%) percentages of the *in vitro*-derived lines were estimated according to the methods reported by Adair (1952). Gelatinization temperature (GT) was determined using alkali spreading method as described by Little et al. (1958). The appearance and disintegration of endosperm were graded visually on the basis of a numerical scale described by Jennings (1979). Furthermore, the average of kernel elongation (%) was determined according to Azeez and Shafi (1966). Amylose content (AC %) was photometrically visualized using a spectrophotometer (Camspec model- M330B, England) according to Williams et al. (1958). AC was further determined using a conversion factor and grouped on the basis of their AC scale as prescribed by Juliano et al. (2009).

### 4.4 Evaluating the resistance of the *in vitro*-derived lines against blast disease

A total of 24 rice blast samples were collected and isolated from different cultivars grown at several Egyptian governorates according to Shabana et al. (2013). Those races were subjected to physiological blast race identification in the rice pathology laboratory and greenhouse of rice research and training center, Sakha, Egypt, according to (Atkins, 1967; Yamada et al., 1976). The *in vitro* rice (INV) lines along with the rice Egyptian popular cultivars were artificially infected with the 24 collected isolates as described (Sehly et al., 2008) under greenhouse condition during the 2024 season. Moreover, the rice genotypes were scanned under open field natural infection at three locations—Kafrelshiekh (Sakha), Gharbia (Gemmiza), and Beheira (Zerzora) governorates—during the 2023 growing season. Under both natural and artificial blast infection conditions, the typical blast lesions were assessed using a 0–9 scale, following the standard evaluation system (Rice, I.N.F.G.E.O, 1996).

### 4.5 Data statistical analysis

Analysis of variance, figure curation, modeling, and genetic analysis of the collected data were generated using the R programing language (Team, R.C, 2000). The packages “lme4”, “dplyr”, “ggplot2”, “factoextra”, “psych”, “agricolae”, and “grid” were employed to fulfill this part.

### 4.6 Marker–trait association *via* single marker analysis

The total genomic DNA of the 24 genotypes was extracted following the CTAB method described by Murray and Thompson (1980). The quantity and quality of the extracted DNA were assessed as described by Abdelrahman et al. (2021). Moreover, 36 SSR markers were used to estimate the genetic diversity among the generated INV lines as well as marker–trait association (**Supplementary Table S10**). The PCR amplification reactions were using 2X GoTaq Green Master Mix (Promega, USA) according to the manufacturer’s recommendation where a 15-µL reaction volume with 7.5 µL GoTaq Master Mix, 2x, 1 µL of forward and reverse primers, 10 uM, 1 µL DNA template, and 4.5 µL nuclease-free water was used. PCR amplification was loaded in 3% agarose gel containing ethidium bromide for electrophoresis in 1X TAE (pH 8.0) using a mini-horizontal electrophoresis system (CBS Scientific, CA, USA). A DNA ladder (100 bp, GeneDirex, Inc., Taiwan) was used for the determination of size of amplicons. The gel was run at 60 V (2.5 V/cm) for 3 h and photographed using Biometra gel documentation unit (BioDoc, Biometra, Germany). The SMA was conducted *via* regression analysis using the R programing language (Team, R.C, 2000). The r_squared_matrix function was used to estimate the linkage between the marker alleles and the phenotypic data at a probability of 0.01. The identified genomic markers that linked to the traits under study were further exposed to allele effect measurement as previously described by Breseghello and Sorrells (2006) (Abdelrahman et al., 2021). The following formula was used to calculate the phenotypic effect value of a single allele:


$$a_{i}=\sum_{j=1}^{ni}x_{ij}/n_{i}-\sum_{j=1}^{nk}x_{kj}/n_{k}$$

where *a_i_* is the phenotypic effect estimate of the allele *i*, *x_ij_* indicates the phenotypic values of *j* variety carrying *i* allele, *n_i_* denotes the number of genotypes carrying *i* allele, *x_k_* depicts the phenotypic value of the variety having the null allele, and *n_k_* represents the number of genotypes carrying the null allele. Alleles with positive effect values were considered as an elite allele if the objective is to increase the value of the trait under study; otherwise, if the objective is to reduce the trait, alleles with a negative effect were considered as elite ones, and carrier accessions are determined accordingly.

## 5 Conclusions

This study successfully developed and evaluated *in vitro*-derived rice lines, identifying AC-2286 and AC-2729 as the highest-yielding genotypes. The predicted genetic gain for grain yield was 0.19 kg m^-^², reflecting a 20.88% improvement over the genotypic mean yield. Several lines exhibited superior grain quality traits, particularly in hulling percentage and head rice recovery. Resistance to multiple physiological races of the blast pathogen was observed in key genotypes, enhancing their suitability for breeding programs. The BLUP model effectively selected top-performing genotypes, AC-2286 and AC-2729, demonstrating its utility in breeding efficiency. Marker–trait association revealed that AC-2729 carries the favorable allele for grain yield while lacks the favorable alleles for blast, while AC-2286 was found to carry several favorable alleles for blast resistance. Our conclusion contributes to accelerating rice breeding programs, improving yield, quality, and disease resistance while supporting the development of resilient rice varieties for sustainable production.

## Data Availability

The datasets generated by this study either presented in the main text or can be found in online repositories. The names of the repository/repositories and accession number(s) can be found in the article/**Supplementary Material**.
