# Supplementary material for: Integrating in vitro breeding, BLUP prediction, and marker analysis to enhance rice yield, quality, and blast resistance
Source: Front Plant Sci. 2025 Sep 16;16:1588427. doi: 10.3389/fpls.2025.1588427 (PMC12502981; doi:10.3389/fpls.2025.1588427)
Supplement: Supplementary file 1 [file Table1.docx]

**Supplementary Table 1.** Average of the vegetative and yield related traits for the tested genotypes

| **Genotype** | **Days to heading**  **(days)** | **Plant height (cm)** | **Panicles number plant^-1^** | **1000-grain weight (g)** | **Yield**  **(kg m^-2^)** |
| --- | --- | --- | --- | --- | --- |
| Giza 178 | 98.67 | 99.40 | 22.67 | 21.63 | 1.03 |
| Giza 177 | 90.67 | 103.33 | 17.67 | 27.47 | 0.85 |
| Sakha 101 | 114.33 | 97.40 | 23.67 | 28.10 | 1.01 |
| Sakha 104 | 99.67 | 105.40 | 23.00 | 26.57 | 1.07 |
| Sakha 108 | 103.33 | 96.93 | 24.00 | 28.19 | 1.07 |
| AC-2286 | 109.00 | 92.40 | 24.33 | 28.46 | 1.10 |
| AC-2301 | 116.67 | 92.27 | 22.00 | 28.00 | 0.98 |
| AC-2420 | 117.33 | 102.20 | 18.33 | 20.27 | 0.77 |
| AC-2423 | 113.00 | 99.80 | 18.33 | 20.36 | 0.85 |
| AC-2491 | 114.33 | 103.93 | 18.00 | 26.17 | 0.95 |
| AC-2530 | 112.33 | 112.80 | 17.00 | 25.57 | 0.93 |
| EM-1803 | 113.67 | 97.13 | 19.00 | 27.33 | 0.95 |
| EM-1816 | 117.33 | 93.87 | 18.33 | 26.10 | 0.84 |
| EM-1881 | 116.33 | 96.27 | 17.33 | 26.47 | 0.74 |
| AC-2541 | 101.00 | 83.53 | 17.33 | 17.67 | 0.89 |
| AC-2593 | 101.67 | 85.87 | 20.67 | 17.97 | 0.90 |
| AC-2561 | 102.67 | 84.60 | 19.67 | 22.63 | 0.88 |
| AC-2656 | 101.67 | 83.93 | 18.67 | 23.63 | 0.77 |
| AC-2677 | 109.00 | 84.40 | 18.67 | 18.83 | 0.82 |
| AC-2709 | 107.00 | 86.07 | 19.33 | 17.87 | 0.81 |
| AC-2712 | 109.33 | 81.27 | 14.67 | 25.23 | 0.83 |
| AC-2724 | 107.33 | 85.00 | 19.67 | 17.63 | 0.75 |
| AC-2729 | 109.33 | 101.47 | 24.00 | 28.50 | 1.11 |
| AC-2806 | 101.67 | 93.67 | 24.00 | 25.87 | 0.89 |
| Mean | 107.81 | 94.29 | 20.01 | 24.02 | 0.91 |

**Supplementary Table 2**: Average of the quality related traits for the tested genotypes

| Genotype | Hulling % | Milling % | Head Rice% | Kernel Elongation % | Gelatinization T. | Amylose Content% |
| --- | --- | --- | --- | --- | --- | --- |
| Giza 178 | 80.44 | 71.06 | 63.22 | 45.45 | 6.33 | 18.38 |
| Giza 177 | 81.67 | 73.06 | 63.22 | 57.17 | 6.33 | 18.43 |
| Sakha 101 | 79.56 | 71.56 | 61.33 | 53.07 | 6.00 | 19.23 |
| Sakha 104 | 81.56 | 71.33 | 63.44 | 55.37 | 6.00 | 19.65 |
| Sakha 108 | 83.56 | 71.95 | 63.22 | 50.83 | 6.00 | 18.59 |
| AC-2286 | 80.22 | 72.17 | 63.11 | 53.95 | 5.33 | 18.27 |
| AC-2301 | 80.00 | 71.04 | 61.78 | 53.99 | 4.33 | 18.43 |
| AC-2420 | 78.00 | 65.17 | 56.56 | 54.14 | 3.67 | 17.32 |
| AC-2423 | 77.44 | 64.78 | 56.13 | 54.00 | 5.67 | 16.81 |
| AC-2491 | 79.44 | 65.17 | 59.89 | 50.09 | 2.00 | 22.46 |
| AC-2530 | 79.11 | 69.61 | 61.11 | 43.47 | 4.00 | 19.90 |
| EM-1803 | 81.33 | 68.50 | 59.33 | 49.86 | 5.00 | 19.41 |
| EM-1816 | 80.22 | 70.00 | 62.11 | 50.38 | 5.67 | 20.12 |
| EM-1881 | 81.33 | 69.61 | 63.44 | 47.54 | 6.00 | 20.00 |
| AC-2541 | 81.55 | 68.62 | 45.49 | 49.79 | 5.67 | 17.21 |
| AC-2593 | 79.22 | 70.72 | 52.11 | 44.53 | 5.33 | 16.95 |
| AC-2561 | 78.33 | 70.05 | 53.89 | 28.68 | 5.67 | 18.28 |
| AC-2656 | 78.33 | 69.98 | 54.35 | 41.38 | 6.00 | 17.58 |
| AC-2677 | 78.67 | 68.83 | 60.22 | 45.39 | 5.00 | 18.66 |
| AC-2709 | 79.11 | 70.72 | 61.33 | 39.66 | 6.00 | 19.63 |
| AC-2712 | 76.89 | 68.61 | 61.11 | 55.48 | 5.33 | 18.78 |
| AC-2724 | 79.00 | 68.50 | 64.00 | 45.22 | 6.00 | 18.14 |
| AC-2729 | 80.22 | 71.83 | 63.22 | 47.44 | 6.00 | 19.04 |
| AC-2806 | 78.00 | 71.83 | 58.67 | 50.41 | 7.00 | 19.92 |
| L.S.D 0.01 | 1.30 | 2.04 | 2.88 | 5.49 | 0.86 | 2.40 |

**Supplementary Table 3**. Analysis of variance for the studied quality characteristics

| Trait | d.f. | Hulling % | Milling % | Head Rice% | Elongation % | Gelatinization T. | Amylose Content% |
| --- | --- | --- | --- | --- | --- | --- | --- |
| Replications | 2 | 0.002 | 1.23 | 3.30 | 10.65 | 0.39 | 0.81 |
| Genotypes | 23 | 7.45** | 14.46^**^ | 61.72^**^ | 120.85** | 3.32** | 4.72^**^ |
| Error | 46 | 0.37 | 0.85 | 1.66 | 6.11 | 0.14 | 1.22 |

** significant at the 0.01 significance level.

**Supplementary Table 4.** *Magnaporthe oryzae* isolates collected from different cultivars and governorates during 2023 and 2024 Seasons

| **Isolate no.** | **Governorate** | **District** | **Rice cultivar** | **Season** |
| --- | --- | --- | --- | --- |
| 1 | Kafrelsheikh | Desouq | Sakha101 | **2023** |
| 2 | Kafrelsheikh | Sakha | Sakha104 | **2023** |
| 3 | Kafrelsheikh | Foaa | Sakha101 | **2023** |
| 4 | Dakahlia | Talkha | Sakha101 | **2023** |
| 5 | Dakahlia | Dekerns | Sakha104 | **2023** |
| 6 | Sharkia | Hehia | Sakha101 | **2023** |
| 7 | Gharbia | EL-Mahala | Sakha104 | **2023** |
| 8 | Gharbia | Qotour | Sakha101 | **2023** |
| 9 | Gharbia | Qotour | Sakha104 | **2023** |
| 10 | Beheira | Elebrahimyia | Sakha104 | **2023** |
| 11 | Kafrelsheikh | Kafrelsheikh | Sakha104 | **2024** |
| 12 | Kafrelsheikh | Desouq | Sakhal01 | **2024** |
| 13 | Kafrelsheikh | Sakha | Sakha108 | **2024** |
| 14 | Dakahlia | Dekerns | Sakha104 | **2024** |
| 15 | Dakahlia | Dekerns | Sakhal01 | **2024** |
| 16 | Dakahlia | Talkha | Sakha108 | **2024** |
| 17 | Sharkia | Ibokabair | Sakha101 | **2024** |
| 18 | Sharkia | Kafrsaker | Sakha104 | **2024** |
| 19 | Sharkia | Zagazig | Sakha104 | **2024** |
| 20 | Gharbia | Gemmiza | Sakha104 | **2024** |
| 21 | Gharbia | Gemmiza | Sakha108 | **2024** |
| 22 | Beheira | Kafreldawar | Sakha101 | **2024** |
| 23 | Beheira | Itaielbarood | Sakha108 | **2024** |
| 24 | Damietta | Kafr Saad | Sakha104 | **2024** |

**Supplementary Table 5.** Rice blast reactions on the international differential varieties (IDVs) under greenhouse artifical inoculation using 24 isolates of *Magnaporthe oryza*

| Isolate no. | IDVs | | | | | | | |  |
| --- | --- | --- | --- | --- | --- | --- | --- | --- | --- |
|  | Raminad str.3 | Zenith | NP-125 | Usen | Dular | Kanto 51 | CI 8970 s | Caloro | Race identification |
| EG 1 | **1** | **1** | **1** | **4** | **1** | **1** | **1** | **1** | ID-16 |
| EG 2 | **1** | **1** | **4** | **4** | **4** | **4** | **3** | **1** | IC-2 |
| EG 3 | **1** | **1** | **4** | **1** | **5** | **4** | **1** | **1** | IC-20 |
| EG 4 | 1 | 1 | **5** | **1** | **1** | **4** | **5** | **7** | IC-25 |
| EG 5 | 1 | 1 | **1** | **7** | **1** | **5** | **1** | **1** | ID-12 |
| EG 6 | 1 | 1 | **1** | **5** | **1** | **5** | **1** | **7** | ID-11 |
| EG 7 | 1 | 1 | **1** | **7** | **1** | **1** | **1** | **7** | ID-15 |
| EG 8 | 1 | 1 | **4** | **5** | **7** | **4** | **7** | **5** | IC-1 |
| EG 9 | 1 | 1 | **1** | **5** | **1** | **5** | **1** | **7** | ID-11 |
| EG 10 | 1 | 1 | **4** | **7** | **4** | **4** | **1** | **7** | IC-3 |
| EG 11 | **1** | **1** | **5** | **4** | **1** | **1** | **1** | **7** | IC-15 |
| EG 12 | **1** | **1** | **1** | **7** | **1** | **5** | **1** | **1** | ID-12 |
| EG 13 | **1** | **1** | **1** | **4** | **1** | **1** | **1** | **1** | ID-16 |
| EG 14 | **4** | **1** | **1** | **4** | **1** | **7** | **7** | **7** | IA-105 |
| EG 15 | **1** | **1** | **5** | **5** | **1** | **1** | **1** | **4** | IC-15 |
| EG 16 | **1** | **1** | **5** | **1** | **1** | **1** | **1** | **1** | IC-32 |
| EG 17 | **1** | **1** | **5** | **1** | **1** | **1** | **1** | **1** | IC-32 |
| EG 18 | **1** | **1** | **1** | **4** | **1** | **4** | **7** | **5** | ID-9 |
| EG 19 | 1 | 1 | **1** | **7** | **1** | **1** | **1** | **7** | ID-15 |
| EG 20 | **1** | **1** | **1** | **1** | **1** | **1** | **1** | **1** | II-1 |
| EG 21 | **1** | **1** | **5** | **4** | **5** | **7** | **1** | **4** | IC-3 |
| EG 22 | 1 | 1 | **1** | **5** | **1** | **5** | **1** | **7** | ID-11 |
| EG 23 | **1** | **1** | **4** | **5** | **4** | **1** | **4** | **7** | IC-5 |
| EG 24 | **1** | **1** | **5** | **4** | **5** | **7** | **1** | **4** | IC-3 |
| **IC%= 50 ID%- 41.6 IA%= 4.17 II% = 4.17** | | | | | | | | | |
| 1-2 = resistant | 3 = moderately resistant | | | 4-6 = susceptible | | | 7-9 = highly susceptible | | |

EG: Egyptian isolate under study; IC, ID, IA and II are the race groups used to categorize *M. oryzae* isolates

**Supplementary Table 6**. Heritability estimates, genetic variability, and selection response for the key agronomic and quality traits in the in vitro-derived rice lines

| **Trait** | **H2** | **S** | **R** | **GCV** | **PCV** | **GA (%)** |
| --- | --- | --- | --- | --- | --- | --- |
| Heading_date_Days | 0.93 | 12.19 | 11.39 | 6.54 | 6.77 | 10.56 |
| Plant_height_cm | 0.78 | 18.11 | 14.18 | 8.74 | 9.88 | 15.04 |
| NPP_panicles | 0.69 | 5.21 | 3.58 | 13.64 | 16.45 | 18.11 |
| GW_1000_g | 0.65 | 4.21 | 2.73 | 7.88 | 9.79 | 10.24 |
| YIELD_Kgm^-2^ | 0.93 | 0.21 | 0.19 | 12.05 | 12.52 | 21.12 |
| Hulling_Percentage | 0.87 | 3.57 | 3.10 | 1.93 | 2.07 | 3.89 |
| Milling_Percentage | 0.84 | 3.52 | 2.95 | 3.06 | 3.34 | 4.24 |
| Head_Rice_Percentage | 0.92 | 4.99 | 4.59 | 7.50 | 7.81 | 7.70 |
| Kernel Elongation Percentage | 0.86 | 9.29 | 7.98 | 12.72 | 13.72 | 16.40 |
| Gelatinization temperature | 0.87 | 1.57 | 1.37 | 18.95 | 20.27 | 25.26 |
| Amylose_Percentage | 0.49 | 3.48 | 1.72 | 5.76 | 8.20 | 9.12 |
| Blast_score | 0.78 | 4.96 | 3.87 | 79.95 | 90.54 | * |

H^2^ : Heritability, S; Selection deferential, R: Genetic gain, GCV: genetic coefficient of variation, PCV: phenotypic coefficient of variation and GA: genetic advance. * this is a categorical data.

Supplementary Table 7. BLUP estimates for the in vitro derived lines for the different agronomic, quality and disease characters

| **Genotype** | **Heading date (Days)** | **Plant height (cm)** | **NPP (panicles)** | **Panicle length (cm)** | **GW 1000 (g)** | **YIELD Kgm-2** | **Hulling (%)** | **Head Rice (%)** | **Elongation (%)** | **G_T** | **AC (%)** | **Blast score** |
| --- | --- | --- | --- | --- | --- | --- | --- | --- | --- | --- | --- | --- |
| AC-2286 | 1.18 | -1.78 | 3.95 | 1.52 | -2.70 | 0.19 | 0.43 | 3.34 | 5.04 | -0.09 | -0.39 | -1.44 |
| AC-2301 | 8.75 | -1.91 | 1.92 | 0.85 | 1.96 | 0.06 | 0.22 | 2.05 | 5.08 | -1.05 | -0.28 | -1.44 |
| AC-2420 | 9.41 | 7.45 | -1.27 | -0.58 | -5.44 | -0.14 | -1.68 | -3.04 | 5.23 | -1.69 | -1.10 | -1.44 |
| AC-2423 | 5.13 | 5.19 | -2.43 | 0.66 | 1.42 | -0.06 | -2.21 | -3.45 | 5.10 | 0.23 | -1.48 | -1.44 |
| AC-2491 | 6.44 | 9.09 | -1.56 | 0.53 | -2.14 | 0.06 | -0.31 | 0.20 | 1.38 | -3.28 | 2.73 | 1.36 |
| AC-2530 | 4.47 | 17.44 | -2.43 | 0.75 | -0.10 | 0.01 | -0.63 | 1.39 | -4.91 | -1.37 | 0.82 | -0.50 |
| AC-2541 | -6.72 | -10.13 | -2.14 | 1.74 | 0.83 | -0.02 | 1.69 | -13.81 | 1.09 | 0.23 | -1.19 | -1.44 |
| AC-2561 | -5.07 | -9.13 | -0.11 | -0.52 | 0.80 | -0.03 | -1.37 | -5.63 | -18.95 | 0.23 | -0.38 | -1.44 |
| AC-2593 | -6.06 | -7.93 | 1.05 | 1.74 | 1.08 | -0.01 | -0.52 | -7.37 | -3.90 | -0.09 | -1.38 | -1.44 |
| AC-2656 | -6.06 | -9.76 | -0.98 | 1.07 | 1.65 | -0.12 | -1.37 | -5.19 | -6.89 | 0.54 | -0.91 | -1.44 |
| AC-2677 | 1.18 | -9.32 | -1.85 | 0.05 | 1.82 | -0.09 | -1.05 | 0.53 | -3.08 | -0.41 | -0.10 | -1.44 |
| AC-2709 | -0.80 | -7.75 | -1.27 | 0.75 | 1.85 | -0.10 | -0.63 | 1.61 | -8.52 | 0.54 | 0.62 | -1.44 |
| AC-2712 | 1.51 | -12.27 | -4.45 | -5.35 | 0.46 | -0.08 | -2.74 | 1.40 | 6.50 | -0.09 | -0.02 | -1.44 |
| AC-2724 | -0.47 | -8.75 | -1.56 | -3.22 | 0.80 | -0.15 | 0.43 | 4.21 | -3.25 | 0.54 | -0.49 | -1.44 |
| AC-2729 | 1.51 | 6.76 | 3.66 | -1.76 | 1.51 | 0.20 | 0.43 | 3.45 | -1.13 | 0.54 | 0.18 | 1.36 |
| AC-2806 | -6.06 | -0.59 | 3.66 | -2.65 | -0.70 | -0.02 | -1.68 | -0.98 | 1.68 | 1.50 | 0.83 | -1.44 |
| EM-1803 | 5.79 | 2.68 | -0.98 | 0.21 | -1.15 | 0.04 | 1.49 | -0.34 | 1.16 | -0.41 | 0.45 | 2.60 |
| EM-1816 | 9.41 | -0.40 | -1.56 | 1.32 | -0.50 | -0.01 | 0.43 | 2.37 | 1.66 | 0.23 | 0.98 | 2.91 |
| EM-1881 | 8.42 | 1.86 | -2.14 | 0.72 | -0.19 | -0.17 | 1.49 | 3.66 | -1.04 | 0.54 | 0.89 | 3.22 |
| Giza 177 | -16.92 | 8.52 | -1.85 | -0.95 | 0.66 | -0.06 | 1.81 | 3.45 | 8.10 | 0.86 | -0.28 | -0.82 |
| Giza 178 | -9.02 | 4.82 | 2.50 | 2.59 | -4.28 | 0.12 | 0.64 | 3.45 | -3.02 | 0.86 | -0.31 | -0.82 |
| Sakha 101 | 6.44 | 2.93 | 3.37 | -0.52 | 1.20 | 0.10 | -0.20 | 1.61 | 4.21 | 0.54 | 0.32 | 4.15 |
| Sakha 104 | -8.03 | 10.47 | 2.79 | -0.33 | -0.10 | 0.15 | 1.70 | 3.66 | 6.39 | 0.54 | 0.63 | 2.60 |
| Sakha 108 | -4.41 | 2.49 | 3.66 | 1.39 | 1.25 | 0.16 | 3.61 | 3.45 | 2.08 | 0.54 | -0.15 | 2.60 |

NPP: number of panicles per plant; GW 1000: 1000 grain weight; G_T: gelatinization temperature; AC: amylose content

Supplementary Table 8.PCA over all ranking of the studied genotypes

| PC1 | PC2 | PC3 | PC4 | PC5 | PC6 | PC7 | PC8 | PC9 | PC10 | PC11 | PC12 | | Genotype | Overall  Rank | |  |
| --- | --- | --- | --- | --- | --- | --- | --- | --- | --- | --- | --- | --- | --- | --- | --- | --- |
| 3.25 | -0.40 | -0.38 | -0.49 | -0.02 | 0.48 | -0.40 | 0.58 | 0.50 | 0.20 | -0.12 | 0.32 | Sakha 104 | | | 1 | |
| 3.23 | -1.42 | 0.17 | -0.33 | -1.10 | 0.36 | 0.05 | -0.41 | -0.41 | 0.64 | -0.04 | 0.25 | Sakha 108 | | | 2 | |
| 2.47 | -0.75 | 0.13 | 0.89 | -0.34 | -0.89 | -0.33 | 0.65 | -0.43 | 0.14 | -0.28 | -0.18 | AC-2729 | | | 3 | |
| 2.30 | 0.04 | 0.75 | 0.05 | -0.91 | -1.36 | 0.61 | 0.81 | 0.37 | -0.55 | 0.11 | 0.43 | Sakha 101 | | | 4 | |
| 1.81 | -0.47 | -1.95 | 0.59 | 0.42 | -1.09 | 0.18 | -1.25 | -0.06 | -0.23 | -0.15 | -0.30 | AC-2286 | | | 5 | |
| 1.54 | -0.90 | -1.91 | 0.68 | 1.50 | 0.84 | 0.92 | -0.15 | -0.15 | 0.15 | -0.58 | -0.08 | Giza 178 | | | 6 | |
| 1.17 | -1.70 | -0.32 | -2.24 | 1.27 | 1.42 | -1.36 | 0.10 | -0.16 | -0.47 | 0.21 | 0.17 | Giza 177 | | | 7 | |
| 0.91 | 1.28 | 1.43 | -0.40 | -0.14 | -0.19 | 0.89 | -0.16 | 0.31 | -0.51 | -0.27 | -0.32 | EM-1816 | | | 8 | |
| 0.90 | 1.40 | 0.37 | -0.26 | -0.93 | 0.62 | 0.72 | -0.21 | 0.03 | 0.01 | -0.35 | -0.12 | EM-1803 | | | 9 | |
| 0.58 | 1.16 | 2.19 | -1.07 | 0.23 | 0.63 | 1.39 | 0.00 | -0.31 | -0.15 | 0.44 | -0.13 | EM-1881 | | | 10 | |
| 0.46 | 0.22 | -0.07 | 0.09 | -0.67 | -1.67 | -1.04 | -0.97 | -0.74 | -0.04 | 0.65 | -0.19 | AC-2301 | | | 11 | |
| 0.43 | 4.67 | 0.15 | 1.39 | -0.34 | 1.10 | -1.12 | -0.43 | 1.04 | 0.46 | 0.10 | 0.28 | AC-2491 | | | 12 | |
| 0.16 | 1.99 | 0.29 | 0.87 | 0.55 | 0.78 | -1.19 | 0.82 | -1.19 | -0.57 | -0.04 | -0.42 | AC-2530 | | | 13 | |
| 0.16 | -1.04 | -0.67 | 0.50 | 1.27 | -0.56 | 0.28 | 0.90 | 1.54 | 0.33 | 0.86 | -0.40 | AC-2806 | | | 14 | |
| -1.14 | -0.99 | 1.67 | 0.70 | 0.94 | 0.02 | -0.17 | -0.26 | -0.05 | 0.11 | 0.18 | -0.31 | AC-2709 | | | 15 | |
| -1.37 | -0.62 | 0.94 | -0.74 | 0.86 | -0.02 | 0.44 | -0.72 | -0.57 | 0.98 | 0.07 | 0.14 | AC-2724 | | | 16 | |
| -1.54 | -1.91 | -0.82 | 0.57 | -0.99 | 0.03 | -0.16 | -0.05 | -0.08 | -0.43 | 0.24 | 0.44 | AC-2593 | | | 17 | |
| -1.80 | -0.15 | 0.94 | 0.13 | 0.19 | -0.49 | -0.49 | -0.51 | -0.07 | 0.27 | -0.05 | 0.24 | AC-2677 | | | 18 | |
| -2.09 | 3.26 | -2.56 | -0.54 | 0.29 | 0.01 | 1.16 | -0.02 | -0.61 | -0.24 | 0.46 | 0.33 | AC-2420 | | | 19 | |
| -2.17 | -1.59 | -0.95 | -0.76 | -2.33 | 1.38 | -0.06 | -0.40 | 0.68 | -0.07 | 0.01 | -0.63 | AC-2541 | | | 20 | |
| -2.21 | -1.77 | 0.59 | 2.40 | 0.01 | 0.77 | 0.45 | 0.32 | -0.30 | -0.10 | -0.27 | 0.16 | AC-2561 | | | 21 | |
| -2.22 | 0.55 | 0.48 | -1.04 | 0.88 | -1.23 | -0.56 | -0.59 | 1.03 | -0.44 | -0.86 | 0.13 | AC-2712 | | | 22 | |
| -2.40 | -1.82 | 0.35 | 0.31 | -0.05 | 0.13 | 0.08 | 0.32 | 0.08 | -0.24 | 0.12 | 0.37 | AC-2656 | | | 23 | |
| -2.44 | 0.98 | -0.82 | -1.28 | -0.59 | -1.07 | -0.30 | 1.62 | -0.44 | 0.74 | -0.43 | -0.20 | AC-2423 | | | 24 | |

**Supplementary Table 9.** Rice blast reactions on the japanese differential varieties (JDVs) and effective gene resistant % under greenhouse test using 24 isolates of *Magnaporthe oryzae*

| **Egyptian Isolate no.** | **JDVs / Target gene** | | | | | | | | | | **Race identification** |
| --- | --- | --- | --- | --- | --- | --- | --- | --- | --- | --- | --- |
|  | **Shin 2** | **Toride 1** | **Tusyake** | **Kanto 51** | **Fukunishiki** | **Ishikarishiroke** | **BL-1** | **Yashiro-ochi** | **Pi No. 4** | **Aichi Asahi** |  |
|  | ***Pik-s*** | ***Piz-t*** | ***Pik-m*** | ***Pik*** | ***Piz*** | ***Pii, PiK-s*** | ***Pib*** | ***Pita*** | ***Pita-2*** | ***Pia*** |  |
| EG 1 | 1 | 1 | 1 | 1 | 1 | 1 | 1 | 1 | 1 | 9 | ID-16 |
| EG 2 | 1 | 1 | 4 | 1 | 7 | 4 | 1 | 1 | 4 | 9 | IC-2 |
| EG 3 | 4 | 1 | 7 | 1 | 7 | 4 | 1 | 1 | 4 | 9 | IC-20 |
| EG 4 | 1 | 1 | 1 | 5 | 1 | 1 | 1 | 1 | 1 | 4 | IC-25 |
| EG 5 | 1 | 1 | 1 | 1 | 1 | 1 | 1 | 1 | 1 | 4 | ID-12 |
| EG 6 | 1 | 1 | 4 | 1 | 1 | 1 | 1 | 1 | 1 | 4 | ID-11 |
| EG 7 | 1 | 1 | 1 | 1 | 1 | 1 | 1 | 1 | 1 | 1 | ID-15 |
| EG 8 | 1 | 1 | 7 | 9 | 1 | 1 | 1 | 1 | 1 | 7 | IC-1 |
| EG 9 | 1 | 1 | 4 | 4 | 1 | 1 | 1 | 1 | 1 | 4 | ID-11 |
| EG 10 | 3 | 1 | 5 | 7 | 1 | 1 | 1 | 1 | 1 | 1 | IC-3 |
| EG 11 | 1 | 1 | 1 | 1 | 1 | 1 | 1 | 1 | 1 | 1 | IC-15 |
| EG 12 | 1 | 1 | 1 | 1 | 1 | 1 | 1 | 1 | 1 | 1 | ID-12 |
| EG 13 | 1 | 1 | 1 | 1 | 1 | 1 | 1 | 4 | 4 | 4 | ID-16 |
| EG 14 | 4 | 4 | 1 | 1 | 1 | 1 | 1 | 4 | 4 | 7 | IA-105 |
| EG 15 | 1 | 4 | 1 | 4 | 1 | 1 | 1 | 4 | 4 | 1 | IC-15 |
| EG 16 | 1 | 4 | 4 | 4 | 4 | 1 | 1 | 4 | 4 | 4 | IC-32 |
| EG 17 | 1 | 4 | 4 | 9 | 4 | 1 | 1 | 4 | 4 | 4 | IC-32 |
| EG 18 | 4 | 9 | 1 | 1 | 1 | 1 | 1 | 9 | 9 | 9 | ID-9 |
| EG 19 | 1 | 7 | 4 | 1 | 1 | 1 | 1 | 7 | 7 | 7 | ID-15 |
| EG 20 | 1 | 1 | 1 | 4 | 1 | 1 | 1 | 1 | 1 | 1 | II-1 |
| EG 21 | 1 | 1 | 7 | 7 | 1 | 1 | 1 | 1 | 1 | 7 | IC-3 |
| EG 22 | 4 | 1 | 7 | 7 | 1 | 1 | 1 | 1 | 1 | 4 | ID-11 |
| EG 23 | 1 | 1 | 4 | 1 | 1 | 1 | 1 | 1 | 7 | 4 | IC-5 |
| EG 24 | 1 | 1 | 7 | 7 | 1 | 1 | 1 | 1 | 7 | 7 | IC-3 |
| **Gene infectivity (%)**% | 83.3 | 75.0 | 41.0 | 54.2 | 83.3 | 91.6 | 100 | 70.8 | 54.2 | 25 | - |
| 1-2 = resistant 3 = moderately resistant 4-6 = susceptible 7-9 = highly susceptible | | | | | | | | | | | |

EG: Egyptian isolate under study; IC, ID, IA and II are the race groups used to categorize *M. oryzae* isolates

Supplementary Table 10. Marker names, chromosome number and sequence of the SSR markers used in the study

| Marker Sequence | | Chromosome number | Marker name |
| --- | --- | --- | --- |
| Reverse | Forward |  |  |
| CAACGAAATCGACACGTTGC | CACTATGCACGTACGCACACC | 3 | RM15338 |
| CAGAAATGGGTGAAGATAGTGAGC | ATGATAGTGTGAAGCCCAACTCC | 3 | RM15578 |
| TGTAGTAGACGAGAGGCCGG | TACTCCTATCCTGCCATGGC | 3 | RM3513 |
| TGCTATAAAAGGCATTCG | ATCGATCGATCTTCACGAGG | 11 | RM 224 |
| GATTCTTCCTCCCCTTCGTG | TTCCCCAATGGAACAGTGAC | 1 | RM1216 |
| TCGGGAAAACCTACCCTACC | ACGGGCAATCCGAACAACC | 8 | RM44 |
| TAGATGAAACACTTGTCGAG | CTGTGTCCTTGTATCAGATG | 1 | RM6887 |
| TAAGTCGATCATTGTGTGGACC | TCTGCAAGCCTTGTCTGATG | 2 | RM208 |
| CATGCTGAAGTAAAACCGGG | AGTACCCTGCCACGGTACAG | 5 | RM3286 |
| GGAGGGAGGAATGGGTACAC | AAACGAGAACCAACCGACAC | 6 | RM1370 |
| TCCTCCTCCACCTCAATCAC | ATTAATACCGCTACCACGCG | 8 | RM6838 |
| AATAGGCCTGTAGTTTTTTC | CAAAGAGCTGATTATGTGTT | 8 | RM5891 |
| CACATTATCTGTCAAGGTCC | CTGGATGAAAGGATACAACA | 10 | RM3773 |
| TTTCTCCCCCCCAACCAC | AACCTGGAGGTGCTGGTCTC | 11 | RM1341 |
| GAACCTACATATCGAGAGCA | TAACGGAGGGAGTAGTTTTC | 12 | RM1986 |
| AGTGTTGTGCGGCACGTGCTTTG | CGCACGCTTTCCGAACTACTCCCGCT | 6 | Z4792 |
| GAGAGGTTTGCAGCCAGACCAGG | CTCAAGATTGTATCGTCGACGACTA | 1 | K 3957 |
| ACAGAATTGACCAGCCAAG | CATGAAAGAAAGGAGTGCAG | 6 | AP5930 |
| CCGGATTCACGAGATAAACTC | GGCTTCATCTTTGGCGAC | 11 | RM286 |
| CGATGTTCGCCATGGCTGCTCC | TCCTCCCTCCCTTCGCCCACTG | 4 | RM131 |
| CATGGATCACCGAGCTCCCCCC | ATCGTCTGCGTTGCGGCTGCTG | 4 | RM124 |
| TGAGCATCCCGTGCTGTC | ACCACCACGCCATTAGAGAC | 8 | RM1345 |
| CACTTGCATAGTTCTGCATTG | CCAGATTATTTCCTGAGGTC | 3 | RM231 |
| CGTCTCCTTTGGTTAGTGCC | GGCTTACTGGCTTCGATTTG | 3 | RM 517 |
| AGCGAAAATCATTTATCACA | TCTACAAACTCAGTTAAACT | 9 | JJ 81 |
| GGATGATGTGATCTGCAGAG | CTCTTGGTGATCTTTGTTAC | 9 | JJ 113 |
| GTAGCCTAGCATGGTGCATG | TCCTTGTGAAATCTGGTCCC | 7 | RM 248 |
| GGTGGCATTCGATTCCAG | TCCAACATGGCAAGAGAGAG | 12 | RM13 |
| TCACCTGGTCAGCCTCTTTC | AGAAGCTAGGGCTAACGAAC | 12 | RM235 |
| CTGATGCTACCAGAATCCTC | CCTCCGTTTCACAATGTAAC | 12 | RM2357 |
| GCCTTCATGCTTCAGAAGAC | CCAATCATTAACCCCTGAGC | 8 | RM404 |
| TGGCCTGCTCTCTCTCTCTC | TAGGACGACCAAAGGGTGAG | 8 | RM515 |
| GATTACTGGTTTGCCATTTG | ATTCATGCTTCCTTTCAGTG | 11 | RM3428 |
