# Supplementary material for: Integrating in vitro breeding, BLUP prediction, and marker analysis to enhance rice yield, quality, and blast resistance
Source: Front Plant Sci. 2025 Sep 16;16:1588427. doi: 10.3389/fpls.2025.1588427 (PMC12502981; doi:10.3389/fpls.2025.1588427)
Supplement: Supplementary file 2 [file DataSheet1.docx]

1. **Materials and Methods**
   1. **F1 hybrids generation**

Five Egyptian rice genotypes, Giza177, Giza179, Sakha101, Sakah104 and GZ9461 were used in this experiment to enhance their yield, quality and blast resistance. Seeds of these five rice genotypes were obtained from the rice nucleus seeds of rice research and training center, Sakha 33717, Egypt. The varieties belonged to the subspecies Japonica type and indica/japonica type. The genotypes IRBL5-M and IR12T127 were crossed with Sakha101 to enhance its resistance to blast, IR83106-B-B-2 used to enhance the yield performance of Giza177, Milyang95 crossed with Giza179 to enhance its quality characteristics, IR12N245 was crossed with GZ9461 to enhance its yield performance, and IRBL7-M was used to enhance blast resistance of the genotype Sakha104. The seeds of the donor genotypes were collected from the genetic stock of rice research and training center, Sakha 33717, Egypt. While the mature embryo of the cultivar Sakha101 was used for embryo culture to develop embryogenesis derived lines, Figure 1.

- 1. **Harvesting the boots at booting stage and cold pre-treatment**

In the subsequent season, F_1_ hybrids were cultivated at the experimental farm of the Rice Research and Training Center in Sakha, Kafrelsheikh, Egypt. During the booting stage, healthy boots were collected from each genotype between 07:00 and 09:00 a.m. These selected boots had a distance of 5–8 cm between the base of the first leaf and the primary leaf node, indicating the optimal stage for microspore collection. At this stage, the anthers contain microspores at the mid-late uninucleate stage, which is conducive to callusing or direct androgenesis (Afza et al., 2000).

The collected boots were first washed under running water after the leaves were trimmed off. They were then wiped with moistened cotton soaked in 70% ethanol to ensure surface sterilization. The sterilized boots were subsequently wrapped in wet cheese cloth and aluminum foil and sealed in polyethylene bags to prevent desiccation and maintain pollen viability. These wrapped boots were then incubated at 8 °C for 7–8 days as a cold pre-treatment.


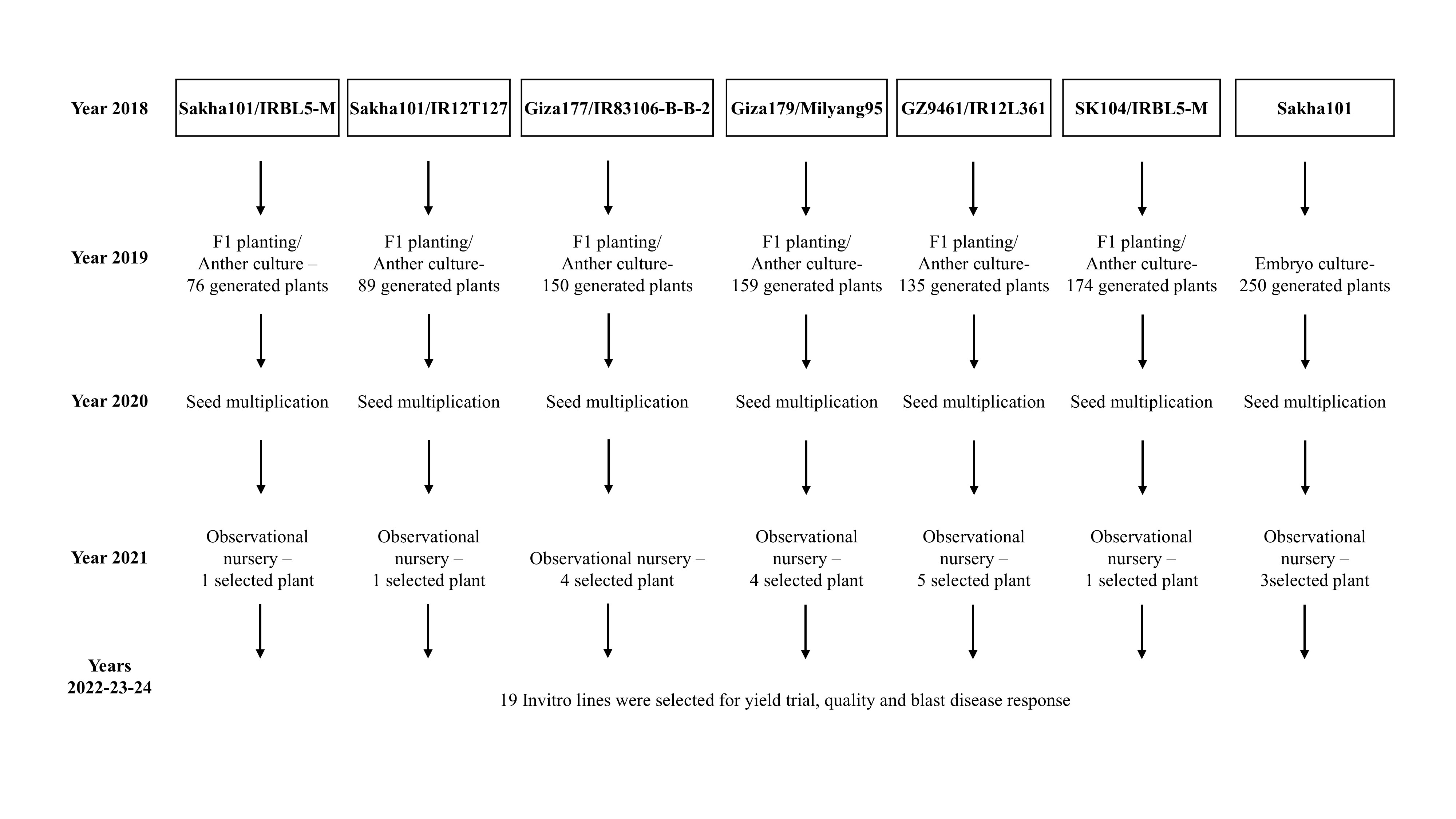


**Figure 1.** Flowchart representing the multi-year breeding and selection process for developing the 19 new rice lines through in-vitro techniques (2018–2023). The breeding scheme started in 2018 with the use of Egyptian parent varieties including Sakha101, Giza177, and GZ9461 and Sakha104 together with the donor varieties IRBL5-M, IR12T127, IR83106-B-B-2, Milyang95 and IR12L361. Various breeding methods (F_1_ planting, anther culture, and embryo culture) were implemented in 2019, resulting in the generation of numerous plants. In 2020, seed multiplication was performed to increase plant populations. In 2021, observational nurseries were conducted to evaluate and select promising plants from each cross. The final step (2022–2023) selected 19 *in vitro*-derived lines were evaluated for yield performance and quality, while blast disease resistance during (2023-2024).

- 1. **Anther and embryo plating and media composition**

The panicles of the hybrids were then collected from the boots and surface sterilized with 70% ethanol for 4 min followed by 2% sodium hypochlorite for 20 min then rinsed three times with sterile double distilled water. Florets were then removed from the sterilized panicles by cutting its base then anthers were platted in Petri dishes (88 mm) with the N6 medium (CHU et al., 1975). This medium was prepared using the commercial N6 medium (Phytotech Laboratories, Shawnee Mission, KS, USA) after adding 30 g L^−1^ sucrose, 1 mg L^−1^ 2,4-D and phytagel 3 gm L^-1^. While for the embryo culture of the cv. Sakha101, the mature embryo was collected from the seeds and sterilized with 70% ethanol for 4 min followed by 2% sodium hypochlorite for 20 min. Sterilized mature embryo was plated in Petri dishes (88 mm) with sterilized MS medium (Murashige and Skoog, 1962). This media was prepared using the commercial MS medium (Sigma-Aldrich, St. Louis, MO, USA) after adding 30 g L^−1^ sucrose, 1 mg L^−1^ 2,4-D and phytagel 3 gm L^-1^. After plating the dishes were incubated at 25°C and complete darkness. The calli (2-3mm) induced in the callus induction media was transferred to regeneration MS medium. Where MS medium supplemented with 3% sucrose, 1 mg L^−1^ kinetin, and 1 mg L^−1^ α-naphthalene acetic acid and 3 g L^−1^ phytagel after adjusting the pH to 5.8. Regeneration media with calli then kept at 25 °C with a relative humidity of 60–85% and a photoperiod of 16 h from white cool fluorescent tubes.

- 1. **Plant Regeneration**

The regenerated green plantlets were then transferred to culture tubes that contained MS medium devoid of any phytohormones to promote root induction. Multiple green plantlets derived from the same calli were individually planted because they might be originated from different pollen grains, which could mean different recombinants. Regenerated plants with roots were incubated for acclimatization on a hydroponic nutrient solution (Yoshida et al., 1971) for 1 week before transplanting in the greenhouse conditions. Plantlets with well-developed roots were then planted in pots in greenhouse conditions for further observation and seed harvesting.

- 1. **Field evaluation for the *in* *vitro*-derived lines:**

The seeds of the DHLs and embryo derived lines were planted under observational nursery for a season based on their field performance 19 lines were selected. These 19 lines along with the most popular cultivars Giza178, Giza177, Sakha101, Sakha104 and Sakha108 being used as check varieties (Supplementary Table 1) were cultivated for the two successive rice growing seasons 2022 and 2023. The field evaluation for these lines conducted at the experimental farm of rice research and training center, Sakha Agricultural Research Station, Kafrelsheikh, Egypt (31°09′ N, 30°9′ E) in randomized complete block design (RCBD) with three replications. Plots were five one-meter-long rows with planting space of 20 cm between transplant and 20 cm between rows. All other cultural practices were conducted as recommended by the Egyptian national rice crop program. *In vitro*-derived lines phenotypic performance was evaluated by measuring; days to heading (days), plant height (cm), number of panicles per plant (panicle), 1000-seed weight (g) and grain yield (g plant^-1^) according to rice standard evaluation system (Rice, 1996).

- 1. **The *in vitro-*derived lines quality characteristics**
     1. **Physical properties of grain quality**

Hulling percentage (%), milling percentage (%) and head rice percentage (%) of the *in* *vitro*-derived lines were estimated, at the grain quality lab, Rice Research and Training Center, Kafrelsheikh, Egypt, according to the methods reported by (Adair, 1952).

Table 1. Source and parents of the genotypes under study:

| **No** | **Entry** | **Source** | **Parentage** |
| --- | --- | --- | --- |
| 1 | Giza 178 | Local Check | Giza175/Milyang 49 |
| 2 | Giza 177 | Local Check | Giza 171/Yomji No.1// Pi No.4 |
| 3 | Sakha 101 | Local Check | Giza 176 / Milyang79 |
| 4 | Sakha 104 | Local Check | GZ 4096-8-1/GZ4100-9-1 |
| 5 | Sakha 108 | Local Check | Sakha101 ∕ HR5824 ∕∕ Sakha101 |
| 6 | INV-1 | Anther culture derived line | SK101/IRBL 5-M |
| 7 | INV -2 | Anther culture derived line | G177/IR83106-B-B-2 |
| 8 | INV -3 | Anther culture derived line | SK101/IR12T127 |
| 9 | INV -4 | Anther culture derived line | SK101/IR12T127 |
| 10 | INV -5 | Anther culture derived line | SK101/IR12T127 |
| 11 | INV -6 | Anther culture derived line | SK101/IR12T127 |
| 12 | INV -7 | Mature embryo culture derived line | EC SK101 |
| 13 | INV -8 | Mature embryo culture derived line | EC SK101 |
| 14 | INV -9 | Mature embryo culture derived line | EC SK101 |
| 15 | INV -10 | Anther culture derived line | G179/milyang95 |
| 16 | INV -11 | Anther culture derived line | G179/milyang95 |
| 17 | INV -12 | Anther culture derived line | G179/milyang95 |
| 18 | INV -13 | Anther culture derived line | G179/milyang95 |
| 19 | INV -14 | Anther culture derived line | GZ9461/IR12N245 |
| 20 | INV -15 | Anther culture derived line | GZ9461/IR12N245 |
| 21 | INV -16 | Anther culture derived line | GZ9461/IR12N245 |
| 22 | INV -17 | Anther culture derived line | GZ9461/IR12N245 |
| 23 | INV -18 | Anther culture derived line | GZ9461/IR12N245 |
| 24 | INV -19 | Anther culture derived line | SK104/IRBL 7-M |

- - - 1. **Hulling percentage (%):**

The seeds were hulled using the experimental huller machine (Satake) and the brown rice % was estimated using equation (1)

Hulling %= $\frac{Brown rice weight(g)}{Rough rice weight (150 g)} \times100$------------------------------------------- Equation 1

- - - 1. **Milling percentage (%):**

The resulted brown rice from the hulling process of the lines and checks under study were milled using Mc GILL Miller No.2. The total milled rice was weighed and calculated as following equation,

Milling % = $\frac{Milled rice weight(g)}{Rough rice weight (150 g)} \times100$------------------------------------------- Equation 2

- - - 1. **Head rice percentage (%):**

Whole milled grains were separated from the total milled rice using a rice sizing device. The separation of these particles is termed as grading. The amount of head rice was obtained and calculated by the following equation:

Head Rice % = $\frac{Whole milled rice weight (g)}{Rough rice weight (150 g)} \times100$------------------------------------ Equation 3

- - 1. **Cooking and eating grain characteristics:**
       1. **Gelatinization temperature (G.T.):**

GT was determined using alkali spreading method described by (Little et al., 1958). GT was estimated as the alkali spreading value (ASV) that is estimated by the extent of dispersal of whole milled rice grains in diluted alkali solution (1.7% KOH). The appearance and disintegration of endosperm were graded visually on the basis of the following numerical scale. A rating of 1 to 3 is classified as high gelatinization temperature (greater than 74˚C), a ratio of 4 to 5 is classified as intermediate gelatinization temperature (70-74˚C) and a rating of 6 to 7 corresponds to gelatinization temperature below 70˚C (Jennings, 1979).

- - - 1. **Kernel elongation (%):**

Using the Micrometer, the length of five rice grains was measured (mm) and their average was determined. Grains were left in Test tube filled with 30 ml of distilled water for 30 minutes, then for another 10 minutes in 98˚C a water tub. After that, the tubes were placed in cold water till reaching room temperature. Grains were lifted from the distilled water, dried (by filter paper), and measured again by graph papers according to (Azeez and Shafi, 1966)

Kernal elongation percentage was valued this formula:

$\frac{Grain Avg.lengt b.c. -Grain Avg.lengta.c.}{Grain Avg.lengt b.c.} \times100$Kernal Elongation %= $\frac{Grain Avg.length b.c. -Grain Avg.length a.c.}{Grain Avg.length b.c.} \times100$ ----------------------- Equation 4

Whereas: b.c.: Before cooking a.c.: After cooking

- - - 1. **Amylose content (AC, %):**

AC was photometrically visualized using spectrophotometer, **(**Camspec model- M33OB, England) according to (Williams et al., 1958). AC was further determined using a conversion factor and grouped on the basis of their AC scale as prescribed by (Juliano et al., 2009).

- 1. **Evaluating the resistance of the *in vitro*-derived lines against blast disease**

The plant material was further tested against blast disease to identify resistant lines. The research work was established in the rice pathology laboratory and greenhouse of rice research and training center, Sakha, Egypt.

- - 1. **Rice blast samples collection and blast fungus isolation**

The rice blast samples were collected from different spots located at different governorates and from different local rice cultivars. These samples were isolated according to (Shabana et al., 2013) as follow. Infected leaves were cut into small pieces of about 1-2 cm and superficially sterilized for 2 min in a 2% sodium hypochlorite solution, and then washed several times with sterile distilled water. Samples were incubated separately for a whole night under fluorescent lighting. On the agar media, the conidia present on the incubated leaves or panicles were spread. The Petri dishes were left for 24 h to germinate the conidium at 24-28ºC. Germinated conidium was picked and transferred to 20% agar. The hyphae tip was cut and transferred onto banana dextrose agar medium. To produce spores, the isolates were cultured and multiplied on a banana medium under fluorescent lighting for 10 days at 28 °C.

- - 1. **Identification of blast physiological races, effective resistant genes and evaluating of rice genotypes under greenhouse conditions**

The *in vitro*-derived lines along with the rice cultivars; Sakha101, Sakha104, Giza177, Giza178 and Sakha108 were evaluated with the 24 collected isolates. According to (Atkins, 1967) eight international differential varieties (IDV) *i.e*., Ramind str 3, Zenith, Np125, Usen, Duler, Kanto5l, She-tiao-tsao(s) and Caloro were used to identify the physiological blast races of the collected isolates. Furthermore, ten international Japanese differential varieties (JDVs) *i.e*., Shin 2 (*Pi-K^s^*), Toride 1 (*Pi-Z^t^*), Tusyake (*Pi*-*K^m^*), Kanto 51(*Pi-K*), Fukunishiki (*Pi-Z*), Ishikarishiki (*Pii*-*Pi-K^s^*), BL-1(*Pib*), Yashiro-Mochi (*Pita*), Pi No. 4 (*Pita*^2^), Aichi Asahi (*Pia*) were used to determine the effective resistant blast genes that corresponds to the collected isolates (YAMADA et al., 1976). Twenty-four isolates were used to inoculate each test genotype. The tested genotypes were seeded in plastic trays (30 × 20 × 15 cm). There were two rows in each of the 15 rows on each (Sehly et al., 2008) The trays were fertilized with urea (46.5% N; 5 g/tray) and housed in the greenhouse at 28±2^o^C. Using an electrical spray gun, spore suspension at a concentration 5×10^5^ spores/ml was inoculated into seedlings at the 3–4 leaf stage. The seedlings were inoculated and kept for 24 h in a moist chamber with more than 90% RH at 28±2^o^C, and then the seedlings were transferred to a greenhouse with comparable conditions.

- - 1. **Evaluation of blast disease under field condition**

Rice genotypes were evaluated under the open field natural infection at three locations; Kafrelshiekh (Sakha), Gharbia (Gemmiza) and Beheira (Zerzora) governorates during 2023 growing season. The typical blast lesions were assessed forty days after sowing using a 0–9 scale, by the standard evaluation system (IRRI, 2013).

- - 1. **Disease severity evaluation**

Using the standard evaluation system's 0–9 scale, blast reactions as well as the typical blast lesions were assessed seven days following inoculation under greenhouse conditions (IRRI, 2013).

- 1. **Genotypic analysis of the *in vitro*-derived lines**
     1. **Genomic DNA isolation**

Total genomic DNA of the 24 genotypes was extracted from their young leaves by crushing them in liquid nitrogen within microfuge tubes, following the CTAB method described by (Murray and Thompson, 1980). The quantity and quality of the extracted DNA were assessed using 0.8% agarose gel electrophoresis, with diluted uncut lambda phage DNA as a size standard in a mini-horizontal electrophoresis system (CBS Scientific, CA, USA). The DNA concentrations were optimized to approximately 30 ηg μl^-1^ for subsequent PCR reaction.

- - 1. **PCR amplification and electrophoresis**

Genetic diversity for 24 genotypes was screening using 36 SSR markers are linked to grain yield, quality and blast resistance. Primers names, sequences and chromosome number are listed in Table 2. The original source, repeat motifs, primer sequences, and chromosomal location can be found on the Gramene website ([http://www.gramene.org](http://www.gramene.org/), accessed on 28 November 2023). PCR amplification reactions were using 2X *GoTaq* Green Master Mix (Promega, USA.) according to the manufacturer recommendation. The reaction mixture was first denatured for 3 min at 94°C for, followed by 35 cycles of denaturation for 30 se. at 94°C, annealing at 55°C for 30 sec. and elongation at 72°C for 30 sec., followed by a final extension at 72°C for 7 min. PCR amplification was loaded in 3% agarose gel containing Ethidium Bromide for electrophoresis in 1X TAE (pH 8.0) using a mini-horizontal electrophoresis system (CBS Scientific, CA, USA). DNA ladder (100bp) was used for determination of size of amplicons. The gel was run at 60 volts (2.5V/cm) for 3 hrs and photographed using Biometra gel documentation unit (BioDoc, Biometra, Germany).

Table 2. Marker names, chromosome number and sequence of the SSR markers used in the study

| Marker Sequence | | Chromosome number | Marker name |
| --- | --- | --- | --- |
| Reverse | Forward |  |  |
| CAACGAAATCGACACGTTGC | CACTATGCACGTACGCACACC | 3 | RM15338 |
| CAGAAATGGGTGAAGATAGTGAGC | ATGATAGTGTGAAGCCCAACTCC | 3 | RM15578 |
| TGTAGTAGACGAGAGGCCGG | TACTCCTATCCTGCCATGGC | 3 | RM3513 |
| TGCTATAAAAGGCATTCG | ATCGATCGATCTTCACGAGG | 11 | RM 224 |
| GATTCTTCCTCCCCTTCGTG | TTCCCCAATGGAACAGTGAC | 1 | RM1216 |
| TCGGGAAAACCTACCCTACC | ACGGGCAATCCGAACAACC | 8 | RM44 |
| TAGATGAAACACTTGTCGAG | CTGTGTCCTTGTATCAGATG | 1 | RM6887 |
| TAAGTCGATCATTGTGTGGACC | TCTGCAAGCCTTGTCTGATG | 2 | RM208 |
| CATGCTGAAGTAAAACCGGG | AGTACCCTGCCACGGTACAG | 5 | RM3286 |
| GGAGGGAGGAATGGGTACAC | AAACGAGAACCAACCGACAC | 6 | RM1370 |
| TCCTCCTCCACCTCAATCAC | ATTAATACCGCTACCACGCG | 8 | RM6838 |
| AATAGGCCTGTAGTTTTTTC | CAAAGAGCTGATTATGTGTT | 8 | RM5891 |
| CACATTATCTGTCAAGGTCC | CTGGATGAAAGGATACAACA | 10 | RM3773 |
| TTTCTCCCCCCCAACCAC | AACCTGGAGGTGCTGGTCTC | 11 | RM1341 |
| GAACCTACATATCGAGAGCA | TAACGGAGGGAGTAGTTTTC | 12 | RM1986 |
| AGTGTTGTGCGGCACGTGCTTTG | CGCACGCTTTCCGAACTACTCCCGCT | 6 | Z4792 |
| GAGAGGTTTGCAGCCAGACCAGG | CTCAAGATTGTATCGTCGACGACTA | 1 | K 3957 |
| ACAGAATTGACCAGCCAAG | CATGAAAGAAAGGAGTGCAG | 6 | AP5930 |
| CCGGATTCACGAGATAAACTC | GGCTTCATCTTTGGCGAC | 11 | RM286 |
| CGATGTTCGCCATGGCTGCTCC | TCCTCCCTCCCTTCGCCCACTG | 4 | RM131 |
| CATGGATCACCGAGCTCCCCCC | ATCGTCTGCGTTGCGGCTGCTG | 4 | RM124 |
| TGAGCATCCCGTGCTGTC | ACCACCACGCCATTAGAGAC | 8 | RM1345 |
| CACTTGCATAGTTCTGCATTG | CCAGATTATTTCCTGAGGTC | 3 | RM231 |
| CGTCTCCTTTGGTTAGTGCC | GGCTTACTGGCTTCGATTTG | 3 | RM 517 |
| AGCGAAAATCATTTATCACA | TCTACAAACTCAGTTAAACT | 9 | JJ 81 |
| GGATGATGTGATCTGCAGAG | CTCTTGGTGATCTTTGTTAC | 9 | JJ 113 |
| GTAGCCTAGCATGGTGCATG | TCCTTGTGAAATCTGGTCCC | 7 | RM 248 |
| GGTGGCATTCGATTCCAG | TCCAACATGGCAAGAGAGAG | 12 | RM13 |
| TCACCTGGTCAGCCTCTTTC | AGAAGCTAGGGCTAACGAAC | 12 | RM235 |
| CTGATGCTACCAGAATCCTC | CCTCCGTTTCACAATGTAAC | 12 | RM2357 |
| GCCTTCATGCTTCAGAAGAC | CCAATCATTAACCCCTGAGC | 8 | RM404 |
| TGGCCTGCTCTCTCTCTCTC | TAGGACGACCAAAGGGTGAG | 8 | RM515 |
| GATTACTGGTTTGCCATTTG | ATTCATGCTTCCTTTCAGTG | 11 | RM3428 |

- - 1. **Single marker analysis (SMA)**

Scoring of amplified bands was done as present (1) or absent (0) for each genotype and primer pair. The resulting allelic data was analyzed against the collected phenotypic data to conduct marker-trait association based SMA. The SMA was conducted via regression analysis using the R programing language (Team, 2000). The r_squared_matrix function was used to estimate the linkage between the marker alleles and the phenotypic data at probability of 0.01. The identified genomic markers that linked to the traits under study were further exposed to allele effect measurement as previously described by (Breseghello and Sorrells, 2006),(Abdelrahman et al., 2021) [. The following formula was used for calculating phenotypic effect value of a single allele:

$a_{i}=\sum_{j=1}^{ni} x_{ij}/n_{i}-\sum_{j=1}^{nk} x_{kj}/n_{k}$-------------------------------------------------------------Equation 4

where *a_i_* is the phenotypic effect estimate of the allele *i*, *x_ij_* indicates the phenotypic values of *j* variety carrying *i* allele, *n_i_* denotes the number of genotypes carrying *i* allele, *x_k_* depicts the phenotypic value of the variety having the null allele and *n_k_* represents the number of genotypes carrying the null allele. Alleles with positive effect values were considered as an elite allele if the objective is to increase the value of the trait under study; otherwise, if the objective is to reduce the trait, alleles with negative effect were considered as elite ones, and carrier accessions are determining accordingly.

References:

Abdelrahman, M., Selim, M.E., Elsayed, M.A., Ammar, M.H., Hussein, F.A., Elkholy, N.K., Elshamey, E.A., Khan, N., and Attia, K.A. (2021). Developing novel rice genotypes harboring specific QTL alleles associated with high grain yield under water shortage stress. *Plants* 10**,** 2219.

Adair, C. (1952). The McGill Miller method for determining the milling quality of small samples of rice.

Afza, R., Shen, M., Zapata-Arias, F.J., Xie, J., Fundi, H.K., Lee, K.-S., Bobadilla-Mucino, E., and Kodym, A. (2000). Effect of spikelet position on rice anther culture efficiency. *Plant Science* 153**,** 155-159.

Atkins, J. (1967). An international set of rice varieties for differentiating races of Piricularia oryzae.

Azeez, M., and Shafi, M. (1966). Quality in rice. Dept. Agr.(W. Pakistan) Tech. *Bull***,** 23.

Breseghello, F., and Sorrells, M.E. (2006). Association mapping of kernel size and milling quality in wheat (Triticum aestivum L.) cultivars. *Genetics* 172**,** 1165-1177.

Chu, C., Chu, W.C., San, S.C., Chen, H., Chu, Y.K., and Yin, C.C. (1975). Establishment of an efficient medium for anther culture of rice through comparative experiments on the nitrogen sources.

Jennings, P.R. (1979). *Rice improvement.* Int. Rice Res. Inst.

IRRI. Standard evaluation system for rice. International Rice Research Institute. 2013. <http://www.knowledgebank.irri.org/images/docs/rice-standard-evaluation-system.pdf>

Juliano, B.O., Perez, C.M., and Resurreccion, A.P. (2009). Apparent amylose content and gelatinization temperature types of Philippine rice accessions in the IRRI Gene Bank. *Philippine Agricultural Scientist* 92**,** 107.

Little, R.R., Hilder, G.B., and Dawson, E.H. (1958). Differential effect of dilute alkali on 25 varieties of milled white rice.

Murashige, T., and Skoog, F. (1962). A revised medium for rapid growth and bio assays with tobacco tissue cultures. *Physiologia plantarum* 15.

Murray, M., and Thompson, W. (1980). Rapid isolation of high molecular weight plant DNA. *Nucleic acids research* 8**,** 4321-4326.

Rice, I.N.F.G.E.O. (1996). *Standard evaluation system for rice.* IRRI, International Rice Research Institute.

Sehly, M., El-Wahsh, S., El-Malky, M., Badr, E., El-Shafey, R., and Aidy, I. (2008). EVALUATION OF CERTAIN EGYPTIAN RICE CULTIVARS TO BLAST DISEASE INCIDENCE DURING FOURTEEN YEARS IN EGYPT. *Journal of Plant Production* 33**,** 2643-2657.

Shabana, Y., El-Wahsh, S., Abdelkhalik, A., Fayzalla, S., and Hassan, A. (2013). Physiological races of rice blast pathogen and host resistant genes under Egyptian conditions. *Journal of Plant Protection and Pathology* 4**,** 709-720.

Team, R.C. (2000). R language definition. *Vienna, Austria: R foundation for statistical computing* 3**,** 116.

Williams, V.R., Wu, W.-T., Tsai, H.Y., and Bates, H.G. (1958). Rice starch, varietal differences in amylose content of rice starch. *Journal of Agricultural and Food chemistry* 6**,** 47-48.

Yamada, M., Kiyosawa, S., Yamaguchi, T., Hirano, T., Kobayashi, T., Kushibuchi, K., and Watanabe, S. (1976). Proposal of a new method for differentiating races of Pyricularia oryzae Cavara in Japan. *Japanese Journal of Phytopathology* 42**,** 216-219.

Yoshida, S., Forno, D.A., and Cock, J.H. (1971). Laboratory manual for physiological studies of rice.
